# Bacterial communities associated with Brassica napus L. grown on trace element-contaminated and non-contaminated fields: a genotypic and phenotypic comparison

**DOI:** 10.1111/1751-7915.12057

**Published:** 2013-04-18

**Authors:** S Croes, N Weyens, J Janssen, H Vercampt, JV Colpaert, R Carleer, J Vangronsveld

**Affiliations:** Hasselt University, Centre for Environmental SciencesAgoralaan Building D, 3590, Diepenbeek, Belgium

## Abstract

Cultivable bacterial strains associated with field-grown *Brassica napus* L. (soil, rhizosphere and roots) from a trace elements (Cd, Zn and Pb) contaminated field and a non-contaminated control field were characterized genotypically and phenotypically. Correspondence analysis of the genotypic data revealed a correlation between soil and rhizosphere communities isolated from the same field, indicating that local conditions play a more important role in influencing the composition of (rhizosphere) soil bacterial communities than root exudates. In contrast, endophytic communities of roots showed a correlation between fields, suggesting that plants on the two fields contain similar obligate endophytes derived from a common seed endophytic community and/or can select bacteria from the rhizosphere. The latter seemed not very likely since, despite the presence of several potential endophytic taxa in the rhizosphere, no significant correlation was found between root and rhizosphere communities. The majority of Cd/Zn tolerant strains capable of phosphorus solubilization, nitrogen fixation, indole-3-acetic acid production and showing 1-aminocyclopropane-1-carboxylate deaminase capacity were found in the rhizosphere and roots of plants growing on the contaminated field.

## Introduction

In our industrialized world, toxic trace elements (TEs) pose a serious concern. Due to atmospheric deposition from four zinc ore smelters in the Dutch-Belgian border region, soils in the Campine region got enriched with cadmium (Cd), zinc (Zn) and lead (Pb) (Sonke *et al*., 2002). Close to the zinc smelters soils contain 3–10 mg Cd per kg dry soil, while at 30 km, background concentrations below 0.5 mg Cd per kg dry soil are found (Koopmans *et al*., 2008). A large portion of this diffusely contaminated region is in agricultural use (Ruttens *et al*., 2010; Witters *et al*., 2011). Increased Cd levels in fodder plants grown on these soils, can lead to increased Cd levels in cattle and hence in the human food chain (Römkens *et al*., 2007). Since Cd is potentially cytotoxic, mutagenic and carcinogenic, farmers are encouraged to remediate their land to ultimately prevent bioaccumulation of toxic TEs in food products (Lim and Schoenung, 2010).

An often suggested remediation strategy for vast areas with diffuse TE contamination is the use of plants and their associated microorganisms to extract TEs from soils and accumulate them in harvestable plant parts (phytoextraction) (Vangronsveld *et al*., 2009). However, low TE availability, uptake, translocation, accumulation and tolerance of plants are still limiting full scale application of phytoextraction (Vangronsveld *et al*., 2009; Weyens *et al*., 2009a). To improve the efficiency of phytoextraction, plant-associated microorganisms with plant growth promoting (PGP) properties and a natural capacity to cope with TEs could be exploited (Weyens *et al*., 2009b). PGP bacteria can stimulate root development (Weyens *et al*., 2011) resulting in an enhanced soil volume explored by roots. Most of the published reviews on PGP bacteria focus on their direct PGP effects in soil (Glick, 2010), rhizosphere (Zhuang *et al*., 2007; Khan *et al*., 2009; Ma *et al*., 2011) and plant (Rajkumar *et al*., 2009; Ma *et al*., 2011). Direct PGP effects can be achieved by the production of phytohormones, 1-aminocyclopropane-1-carboxylate (ACC) deaminase and siderophores, by nitrogen fixation and phosphates solubilization. Plant-associated bacteria that produce siderophores and/or organic acids can also enhance TE availability in soils and by consequence their uptake by plants (Li and Wong, 2010; Rajkumar *et al*., 2010). To assist their host plants to cope with these increased amounts of TEs, endophytic bacteria equipped with a TE sequestration system are of special interest since they can reduce phytotoxicity and increase TE translocation to aerial plant parts (Lodewyckx *et al*., 2001; Sessitsch and Puschenreiter, 2008). Exploiting these bacterial skills, plants with higher biomass and increased tolerance to TEs can be obtained, eventually resulting in a more efficient phytoextraction.

We investigate *Brassica napus* L. (rapeseed) as a candidate phytoextraction crop because it combines high biomass production with a good tolerance to Cd and Zn (Marchiol *et al*., 2004). At the same time it is a valorizable oil producing crop (Vangronsveld *et al*., 2009), mainly used in food applications and biofuel production (Grispen *et al*., 2006). Combining phytoextraction and biofuel production sounds economically attractive, especially since oil prices are increasing and environmental standards are high (Stephenson *et al*., 2008). Moreover, oil and seed meal with acceptable Cd and Zn concentrations could be used to enrich fodder with carbohydrates, proteins and phytosterols (Gül and Şeker, 2006; Iqbal *et al*., 2008). Combining the phytoextraction and economic potentials of *B. napus* could become the decisive factor for a successful remediation in diffusely contaminated areas, like the Campine region, especially when rapeseed-associated bacteria could enhance Cd phytoextraction efficiency.

Since the natural habitat is considered as an interesting model for the evolution of TE tolerant PGP microorganisms (Ma *et al*., 2009), we characterized the cultivable bacterial communities associated with bulk soil, rhizosphere soil and roots of *B. napus* grown on a Cd, Zn and Pb-contaminated field in Lommel [Belgium; trace element field (TE-F)] and a non-contaminated field in Alken [Belgium; control field (CO-F)]. The main objectives of this study were to extend our knowledge on the poorly known bacterial communities associated with *B. napus* and to identify PGP, Cd tolerant and Cd solubilizing bacteria which might increase biomass production and Cd uptake by rapeseed growing under the unfavourable environmental conditions occurring on contaminated fields.

## Results

### Isolation of *B. napus*-associated bacteria

Bacteria were isolated from bulk soil, rhizosphere soil and roots of *B. napus*, grown on an uncontaminated control field (CO-F) and a contaminated field (TE-F) (Table 1).

**Table 1:** Mean total numbers of colony-forming units (cfu) per gram fresh weight of the compartments (COMPT) bulk soil (BS), rhizosphere soil (RS) and *B. napus* root tissue (R) isolated on the control field (CO-F) and the contaminated field (TE-F)

Field	COMPT	cfu g^−1^ fresh weight
CO-F	BS (ac)	99.7 × 10^5 ^± 51.8 × 10^5^ (6)
	RS (b)	23.7 × 10^8 ^± 21.2 × 10^8^ (17)
	R (ac)	10.4 × 10^5 ^± 23.4 × 10^4^ (15)
TE-F	BS (a)	20.5 × 10^6 ^± 22.4 × 10^5^ (20)
	RS (b)	78.1 × 10^7 ^± 30.8 × 10^7^ (25)
	R (c)	22.4 × 10^5 ^± 19.8 × 10^5^ (18)

Values are mean ± standard error of three biological independent replicates. Numbers of different bacterial genera are marked between parentheses. Letters between parentheses in the COMPT column refer to statistical significances in cfu g^−1^ fresh weight (*P*-value < 0.10).

For both fields, the number of cultivable strains recovered from the bulk soil and roots were significantly lower compared with the rhizosphere soil. Significantly more cultivable strains were isolated from the bulk soil compared with the roots at the contaminated field. Considering compartments, no differences in the amount of isolated strains between fields were observed. At both fields, the number of different bacterial genera was higher in the rhizosphere soil than in bulk soil and root.

### Genotypic characterization

**Figure 1:**
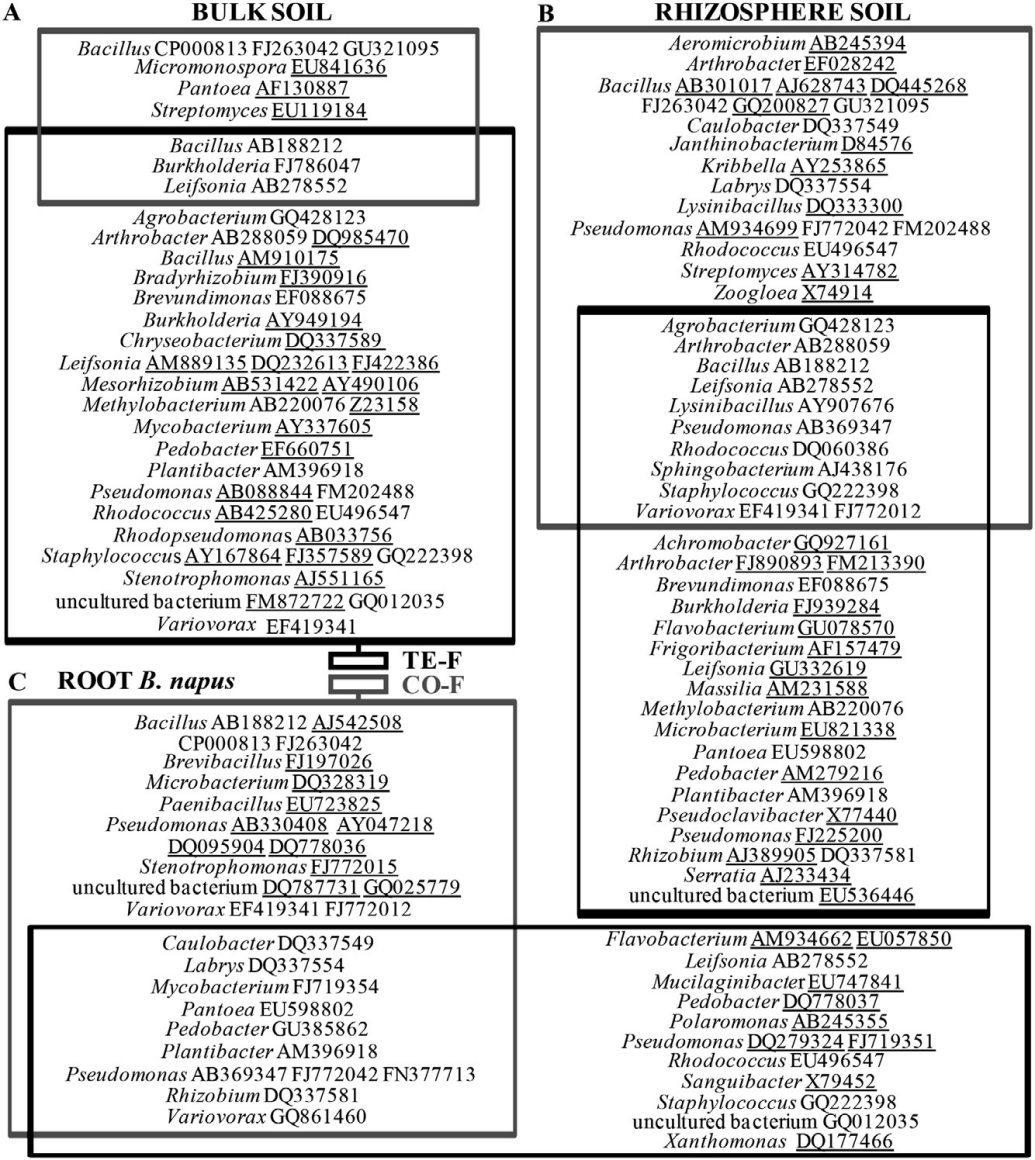
Diversity of cultivable bacterial strains isolated from bulk soil (A), rhizosphere soil (B) and *B. napus* root samples (C) taken at the control field (CO-F) and the contaminated field (TE-F). Bacterial strains present in the intersections were found at both fields, underlined strains were exclusively found in that specific compartment. Behind each bacterial genus, different accession numbers are represented (see Appendix S1 for abundances).

After isolation and purification, five purified replicates of all morphologically different bacterial strains isolated from bulk soil, rhizosphere soil and roots of *B. napus* were characterized by amplified 16S rDNA restriction analysis (ARDRA) using the restriction enzyme HpyCH4IV. One representative member of all strains with identical fingerprints was sequenced for identification by means of Sequence Match at the Ribosomal Database Project II. All (except *Chryseobacterium* DQ337589) strains have a sequence match score higher than 0.900, which indicates a confident identification to the genus level (Appendix S1). Also the neighbour-joining tree clustered strains belonging to the same genus together, confirming the results of the 16S rRNA genes-based identification procedure (Appendix S2). The identification resulted in 37 different bacterial genera recovered from the contaminated field and 29 from the control field (Fig. 1).

**Figure 2:**
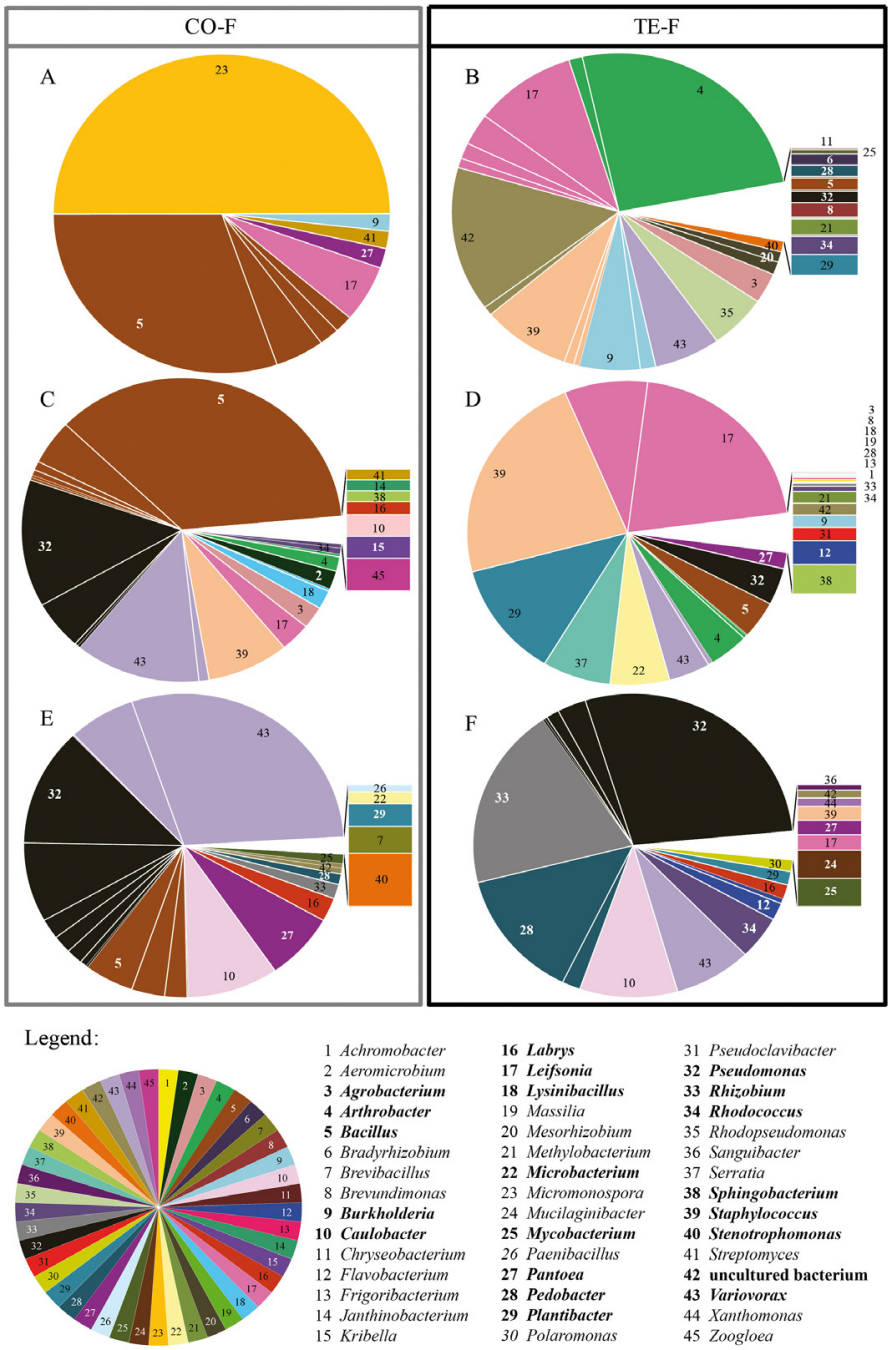
Diversity and abundance of cultivable bacterial strains isolated from bulk soil, rhizosphere soil and root samples taken at the control field (CO-F) (A, C and E respectively) and the contaminated field (TE-F) (B, D and F respectively). Each colour (number) represents a bacterial genus, subdivided colours represent bacterial genera with different accession numbers. Pie fragments indicate the relative abundance, expressed in percentages (see Appendix S1), of the total number of cultivable bacteria isolates per gram fresh weight that are present in the bulk soil, rhizosphere soil and inside the roots of *B. napus.* Bacterial strains with abundances lower than 1% (percentage shown between parentheses) are shown separately next to the pie diagram. Bacterial genera which are marked bold in the legend were found at both fields.

Eighteen of the identified genera were found at both fields. To visualize the diversity and abundance of cultivable *B. napus*-associated bacteria, pie diagrams were prepared (Fig. 2). Each colour (number) represents a bacterial genus; subdivided colours represent genera with different accession numbers. The relative abundance of each genus was expressed as a percentage of the total number of cultivable isolates per gram fresh weight bulk soil, rhizosphere soil and roots (Table 1).

The cultivable soil bacteria at the control field were dominated by the genera *Micromonospora* (50%), *Bacillus* (38.7%) and *Leifsonia* (5.8%) and at the contaminated field by *Arthrobacter* (26.9%), *Leifsonia* (15.7%), *Staphylococcus* (10.0%), *Burkholderia* (7.4%), *Variovorax* (6.4%) and *Rhodopseudomonas* (5.6%). The major part of the cultivable rhizosphere bacteria at the control field consisted of *Bacillus* (43.2%), *Pseudomonas* (19.3%), *Variovorax* (13.7%) and *Staphylococcus* (8.3%) and at the contaminated field of *Leifsonia* (29.3%), *Staphylococcus* (22.7%), *Plantibacter* (12.3%), *Serratia* (6.9%) and *Microbacterium* (6.0%). *Variovorax* (34.5%), *Pseudomonas* (29.4%), *Bacillus* (12.4%), *Caulobacter* (8.6%) and *Pantoea* (6.5%) dominated the cultivable root endophytes at the control field, while at the contaminated field it were *Pseudomonas* (33.0%), *Rhizobium* (16.1%), *Caulobacter* (14.9%), *Pedobacter* (14.7%) and *Variovorax* (7.8%) (Fig. 2).

The number of genotypically different bacterial strains occurring in the same compartment at both fields (see intersections) increases from bulk soil to rhizosphere soil and roots (Fig. 1). Bacterial strains present at both fields in the bulk soil were *Bacillus* (AB188212), *Burkholderia* (FJ786047) and *Leifsonia* (AB278552); in the rhizosphere it were *Agrobacterium* (GQ428123), *Arthrobacter* (AB288059), *Bacillus* (AB188212), *Leifsonia* (AB278552), *Lysinibacillus* (AY907676), *Pseudomonas* (AB369347), *Rhodococcus* (DQ060386), *Sphingobacterium* (AJ438176), *Staphylococcus* (GQ222398) and *Variovorax* (EF419341, FJ772012); and in the roots it were *Caulobacter* (DQ337549), *Labrys* (DQ337554), *Mycobacterium* (FJ719354), *Pantoea* (EU598802), *Pedobacter* (GU385862), *Plantibacter* (AM396918), *Pseudomonas* (AB369347, FJ772042, FN377713), *Rhizobium* (DQ337581) and *Variovorax* (GQ861460).

Bacterial strains exclusively occurring in one of the investigated compartments at the control respectively the contaminated field are underlined in Fig. 1. From the strains shown in the intersection (= occurring at both fields) of Fig. 1C, only *Mycobacterium* (FJ719354), *Pedobacter* (GU385862), *Pseudomonas* (FN377713) and *Variovorax* (GQ861460) were exclusively found in the roots. *Pantoea* (EU598802), *Plantibacter* (AM396918) and *Rhizobium* (DQ337581) were isolated from roots at both fields; at the control field they were not found in bulk neither rhizosphere soil, like at the contaminated field *Caulobacter* (DQ337549), *Labrys* (DQ337554) and *Pseudomonas* (FJ772042) did not occur in other compartments. Other root endophytes were also found in bulk and/or rhizosphere soil [CO-F: *Bacillus* (AB188212), *Bacillus* (FJ263042); TE-F: *Leifsonia* (AB278552), *Plantibacter* (AM396918), *Staphylococcus* (GQ222398)]. Bacterial strains restricted to the bulk and rhizosphere soil at the control field were *Bacillus* (GU321095) and *Leifsonia* (AB278552) and at the contaminated field *Agrobacterium* (GQ428123), *Arthrobacter* (AB288059), *Bacillus* (AB188212), *Brevundimonas* (EF088675), *Methylobacterium* (AB220076) and *Variovorax* (EF419341).

**Figure 3:**
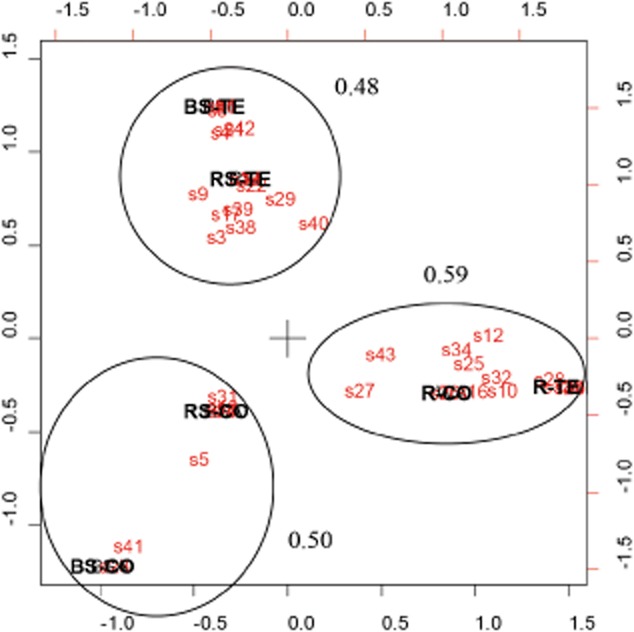
Correspondence analysis of bacterial communities isolated from bulk soil, rhizosphere soil and *B. napus* root samples taken at the control field and the contaminated field. Each number (s1–s45) represents an isolated bacterial genus, the connection between genera and numbers can be found in the legend of Fig. 2. Clustered compartments point out the correlation between the bacterial communities found in the bulk soil, rhizosphere soil and roots collected at the control field (BS-CO, RS-CO and R-CO respectively) and the contaminated field (BS-TE, RS-TE and R-TE respectively). Correlation coefficients of clustered compartments are indicated.

Based on the correspondence analysis (CA) of these data (Fig. 3), we can conclude that the bacterial communities isolated from bulk and rhizosphere soil at both fields are correlated within field [correlation coefficients: 0.48 (TE-F) and 0.50 (CO-F)]. Also root bacterial communities at both fields are correlated (correlation coefficient: 0.59).

### Phenotypic characterization

#### TE tolerance

All purified isolates were screened for their TE tolerance (Table 2A). Percentages of strains from the rhizosphere and from the roots tolerant to 0.8 mM Cd were similar at both fields. At the contaminated field, higher percentages of bulk soil bacteria were tolerant to 0.8 and 1.6 mM Cd, also the rhizosphere soil and the roots contained much higher percentages of strains tolerant to 1.6 mM Cd compared with the control field. The highest percentages of Cd tolerant strains at the contaminated field were found in the roots, while at the control field highest Cd tolerance was occurring in the rhizosphere and roots. Contaminated bulk and rhizosphere soil contained higher percentages of Zn tolerant strains for all tested Zn concentrations (0.6, 1.0 and 2.5 mM Zn). Roots from both fields harboured similar percentages of 0.6 mM Zn tolerant bacteria, while percentages of root strains tolerant to 1.0 and 2.5 mM Zn were higher at the contaminated field. The highest percentages of Zn tolerant strains at the contaminated field were found in the rhizosphere soil, while at the control field this was in the roots. The two-way analysis of variance (anova) test indicated a significant field effect especially when considering tolerance to the highest Cd and Zn concentrations (*P*-values: 0.03 and 0.00007 respectively).

**Table 2:** Phenotypic characterization of all purified bulk soil, rhizosphere soil and *B. napus* root isolates collected at the control field (BS-CO, RS-CO and R-CO respectively) and the contaminated field (BS-TE, RS-TE and R-TE respectively)

	BS-CO	BS-TE	RS-CO	RS-TE	R-CO	R-TE
A						
Cd (0.8 mM)	1.8 ± 1.8	10.8 ± 5.9	14.5 ± 7.4	13.1 ± 6.6	28.1 ± 11.7	22.7 ± 3.2
Cd (1.6 mM)	1.8 ± 1.8	9.2 ± 5.8	3.0 ± 3.0	12.5 ± 6.4	2.2 ± 2.0	14.7 ± 6.3
Zn (0.6 mM)	25.8 ± 25.8	95.3 ± 2.0	43.1 ± 28.5	99.2 ± 0.8	66.8 ± 14.4	52.2 ± 17.5
Zn (1.0 mM)	10.5 ± 10.5	61.0 ± 6.2	2.4 ± 1.7	71.5 ± 13.4	17.6 ± 7.7	32.8 ± 12.0
Zn (2.5 mM)	8.8 ± 8.8	58.0 ± 5.8	1.1 ± 0.6	65.5 ± 10.2	16.7 ± 8.0	32.6 ± 11.8
B						
SID	21.2 ± 21.2	52.6 ± 8.0	79.1 ± 10.6	26.3 ± 7.3	80.8 ± 6.1	41.5 ± 26.8
OA	6.0 ± 6.0	4.9 ± 2.9	39.8 ± 29.9	23.6 ± 12.0	10.6 ± 8.1	19.3 ± 15.5
ACC	0.0 ± 0.0	39.2 ± 8.3	18.3 ± 12.9	37.1 ± 12.9	33.1 ± 13.1	37.3 ± 22.3
IAA	26.8 ± 26.8	32.9 ± 2.5	31.7 ± 20.7	36.4 ± 6.9	36.4 ± 12.3	61.5 ± 12.6
Acetoin	17.3 ± 17.3	2.8 ± 1.6	49.2 ± 25.3	8.6 ± 8.6	17.0 ± 5.3	2.5 ± 2.5
P sol	11.6 ± 11.6	44.3 ± 3.2	19.6 ± 10.8	58.1 ± 5.2	50.1 ± 3.4	49.2 ± 24.5
N_2_ fix	2.0 ± 2.0	3.7 ± 2.3	6.2 ± 6.1	30.4 ± 9.0	8.7 ± 3.2	10.8 ± 7.8

Data are shown as relative abundances, expressed in percentages, of the total number of cultivable bacterial isolates per gram fresh weight bulk soil (BS), rhizosphere soil (RS) and roots (R) at both fields which were tolerant to different concentrations of Cd (0.8 and 1.6 mM) and Zn (0.6, 1.0 and 2.5 mM) (A); and were capable of phosphorus solubilization (P sol), nitrogen fixation (N_2_ fix) and the production of siderophores (SID), organic acids (OA), ACC deaminase (ACC), indole-3-acetic acid (IAA) and acetoin (B). Values are mean ± standard error of three biological independent replicates.

#### PGP characteristics

All strains were screened for their potential PGP characteristics (Table 2B). The percentage of siderophore producing strains in the bulk soil was more than two times higher at the contaminated field, while in the rhizosphere and roots more siderophore producing strains were present at the control field. At both fields similar low percentages of organic acid producing strains were isolated from the bulk soil, while rhizosphere soil and root samples from the control field contained higher respectively lower percentages of these bacteria compared with the contaminated field. All compartments at the contaminated field contained higher percentages of ACC deaminase producing strains as compared with the control field. Moreover similar percentages were found in all compartments at the contaminated field while percentages increased from bulk soil to the root at the control field. Phosphorus solubilization capacity showed a similar distribution pattern. The relative abundance of indole-3-acetic acid (IAA) producing strains was similar in the bulk and rhizosphere soil from both fields and in the control roots, while proportionally twice as much IAA producing strains were isolated from roots at the contaminated field. The percentages of acetoin producing bacterial strains at the control field were higher in all studied compartments; at the contaminated field this was the case for nitrogen fixating strains.

The highest percentages of strains able of solubilizing phosphorus, fixating nitrogen and producing siderophores, ACC deaminase and IAA at the control field were found in the roots, while proportionally the most organic acid and acetoin producing strains were detected in the rhizosphere soil. At the contaminated field, percentages of siderophore and ACC deaminase producing strains were highest in the bulk soil, while percentages of phosphorus solubilizing, nitrogen fixating and organic acid/acetoin producing strains were proportionally highest in the rhizosphere soil. The most IAA producing strains at the contaminated field were found inside the roots. The two-way anova test indicated a significant field effect considering phosphorus solubilization, nitrogen fixation and siderophore and acetoin production (*P*-values: 0.05, 0.06, 0.08 and 0.05 respectively).

### TE concentrations in soils and plants

Total TE concentrations were determined in bulk soil and plant parts (root, stem, leaf and seed) (Table 3). In addition, plant available TE concentrations in bulk soil were estimated using a Ca(NO_3_)_2_ selective extraction.

**Table 3:** Soil and plant trace element (TE) concentrations; Ca(NO_3_)_2_-extractable (*extr*) essential (Zn, Cu, Fe) and non-essential (Cd, Pb) TE concentrations [mg (kg dry weight)^−1^] measured in bulk soil and total essential and non-essential TE concentrations [mg (kg dry weight)^−1^] in bulk soil and *B. napus* plants (root, stem, leaf and seed) from the control field (CO-F) and the contaminated field (TE-F)

	Bulk soil (*extr*)		Bulk soil (*total*)		Root		Stem		Leaf		Seed	
	Mean	Error	Mean	Error	Mean	Error	Mean	Error	Mean	Error	Mean	Error
	CO-F											
Cd	0.15	0.0073	0.50	0.00	0.29	0.054	0.22	0.021	0.40	0.012	nd	nd
Zn	4.6	0.023	89	4.6	33	7.4	22	3.6	77	4.0	39	0.89
Pb	nd	nd	25	0.85	nd	nd	nd	nd	nd	nd	nd	nd
Cu	0.20	0.0073	15	0.41	3.7	1.1	nd	nd	nd	nd	9.5	4.8
Fe	1.8	0.98	9141	223	354*	97	27	3.7	88	10	47	1.4
	TE-F											
Cd	1.0****	0.0033	5.1****	0.088	3.1***	0.32	4.6***	0.31	7.2**	0.45	0.88	0.081
Zn	78****	0.48	277****	6.7	490***	35	472****	27	863***	70	96****	1.5
Pb	0.38	0.010	199****	2.7	8.5	0.63	nd	nd	3.4	0.20	nd	nd
Cu	0.18	0.0050	27****	0.23	3.2	0.074	nd	nd	3.6	0.22	4.8	0.27
Fe	0.58	0.035	2209****	64	43	2.6	18	0.28	89	6.3	76****	3.2

Values are mean ± standard error of three biological independent replicates. Trace element concentrations in soil and plant compartments were compared between fields (significance levels:

* *P* < 0.05;

** *P* < 0.01;

*** *P* < 0.001;

**** *P* < 0.0001). Trace element contents which were too high or too low to be detected are indicated by ‘saturated’ (sat) and ‘not detected’ (nd) respectively.

Amounts of both total and Ca(NO_3_)_2_-extractable Cd, Zn and Pb in the bulk soil were significantly higher at the contaminated field as compared with the control field, like also the Cd and Zn concentrations measured in the roots, stems, leaves and seeds. Lead concentrations in plant parts were below detection limit while total and Ca(NO_3_)_2_-extractable Pb concentrations in the soil at the contaminated field were significantly higher than at the control field. Plants grown at the contaminated field contained significantly more Cd and Zn in their leaves compared with the roots and stems, their seeds accumulated acceptable Cd concentrations (< 1.14 mg Cd kg^−1^) according to the European standards for animal feed (Appendix S3). At both fields, plants contained adequate tissue levels of Ca, K, Mg, Na and Zn (data not shown). The Cu and Fe concentrations in plant tissues were just below the prescribed levels for adequate growth but are not expected to be limiting. Total bulk soil Cu and Fe concentrations were significantly higher at the contaminated field whereas the Ca(NO_3_)_2_-extractable concentrations were similar at both fields. When plant parts were compared between fields, total Fe concentrations were significantly higher in roots from the control and seeds from the contaminated field.

## Discussion

Agricultural soils in the Campine region are diffusely contaminated with Cd, Zn and Pb (Table 3). Since the contamination is due to past atmospheric deposition of these TEs from Zn smelters it is concentrated in the upper soil layer (Sonke *et al*., 2002). Therefore, accumulating plant species developing their root system in this soil layer might be interesting candidates for phytoextraction in this region. An ideal plant for TE phytoextraction should produce high biomass and should take up and translocate to its shoots a significant part of the TEs of concern (Kärenlampi *et al*., 2000). Since the calculated time periods for phytoextraction of toxic TEs are long, it is necessary that the selected crop can be valorized (Vassilev *et al*., 2004; Vangronsveld *et al*., 2009; Meers *et al*., 2010).

*Brassica napus*, a high biomass oil delivering crop, has a good potential to meet most of these criteria. Indeed, roots of *B. napus* are abundantly developing in the upper soil layer (Marchiol *et al*., 2004). Moreover, rapeseed translocates assimilated TEs to its leaves at the end of the growing season (Table 3 and Appendix S3). In case rapeseed can be used as a crop for phytoextraction of toxic TEs, farmers can make profit out of the cultivation by valorizing the seeds (biofuel versus fodder) and other plant parts (biogas). Rapeseed oil can be utilized as alternative fuel whereas intact seeds can be used as animal fodder. Seeds of plants grown on the contaminated field contained acceptable Cd and Pb concentrations (< 1.14 mg Cd kg^−1^ and < 11.4 mg Pb kg^−1^) according to the European standards for animal feed, unlike the other plant parts harvested on the contaminated field (Table 3 and Grispen *et al*., 2006). Fermentation processes can be adopted to reduce the quantity of contaminated biomass while producing biogas (Van Ginneken *et al*., 2007).

However, the amounts of toxic TEs that can be extracted by *B. napus* are still much too low to allow significant reductions of these TE contents in the soil in realistic periods of time (Vangronsveld *et al*., 2009). To improve the applicability and efficiency of phytoextraction, plant-associated bacteria could be exploited to enhance biomass production and to increase TE availability, uptake, translocation and tolerance of plants (Weyens *et al*., 2009a,2009b). Therefore, we investigated the diversity of cultivable bacteria associated with *B. napus* from plants grown on a non-contaminated (control) and a contaminated field as well as the characteristics of the isolated bacterial communities that might contribute to improve biomass production and TE uptake and translocation. Approximately 500 morphologically different bacterial strains were isolated from bulk soil, rhizosphere soil and roots of *B. napus* at both fields and identified based on 16S rDNA sequencing (Figs 1 and 2).

The higher amount of cultivable bacteria in the rhizosphere than in bulk soil (Table 1) can be explained by the ‘rhizosphere effect’ (Rouatt *et al*., 1960). Growth and activity of soil microorganisms are mainly limited by organic carbon (Demoling *et al*., 2007). Poor decomposability of soil organic matter in contrast with easily decomposable root exudates results in higher microbial density/diversity in the rhizosphere (Soderberg and Bååth, 1998). The numbers of cultivable rhizosphere bacteria and root endophytes are in accordance with literature (Benizri *et al*., 2001; Hallmann, 2001); bacterial density/diversity decreased from the rhizosphere to the roots (Table 1 and Fisher *et al*., 1992). The rapeseed-associated bacterial populations that we characterized were dominated by *Bacillus*, *Pseudomonas*, *Variovorax*, *Leifsonia*, *Micromonospora*, *Staphylococcus*, *Arthrobacter* and *Caulobacter* (Fig. 2). Some of these genera were already reported in earlier studies on rapeseed-associated populations (see below), while others are mentioned for the first time in our study. This might be due to (i) differences in isolation protocols (Siciliano and Germida, 1999), growth media and identification procedures (Germida and Theoret, 1997; Kaiser *et al*., 2001) and (ii) the environmental growing conditions of the plants (Lemanceau *et al*., 1995; Song *et al*., 1999).

Germida and colleagues (1998) and Siciliano and Germida (1999) were the first to investigate bacterial communities associated with field-grown *B. napus* using fatty acid methyl ester (FAME) profiles, a tentative identification method (Haack *et al*., 1994). They concluded that the rhizosphere and root interior were colonized mainly by the genera *Bacillus*, *Flavobacterium*, *Micrococcus*, *Rathayibacter*, *Pseudomonas*, *Variovorax* and *Arthrobacter*. Larcher and colleagues (2008) isolated similar genera from the rhizosphere (*Serratia*, *Stenotrophomonas*, *Microbacterium*, *Paenibacillus*, *Arthrobacter*, *Variovorax* and *Pseudomonas*) and roots (*Serratia*, *Pseudomonas*, *Stenotrophomonas* and *Microbacterium*) of field-grown *B. napus*. Additionally, Kaiser and colleagues (2001) and Granér and colleagues (2003) demonstrated that greenhouse and field grown *B. napus* showed corresponding genera in the rhizosphere, root, stem and/or seed, including *Agrobacterium*, *Paenibacillus*, *Bacillus*, *Pseudomonas*, *Chryseobacterium*, *Pantoea*, *Caulobacter*, *Variovorax*, *Stenotrophomonas*, *Arthrobacter*, *Microbacterium*, *Streptomyces* and *Staphylococcus*.

Common bacterial genera identified during our research and not yet mentioned in earlier work on *B. napus* are *Burkholderia*, *Labrys*, *Leifsonia*, *Lysinibacillus*, *Micromonospora*, *Mycobacterium*, *Pedobacter*, *Plantibacter*, *Rhizobium*, *Rhodopseudomonas* and *Sphingobacterium* (Fig. 2). Many bacterial strains appeared in both soil compartments (Fig. 1) and based on the correspondence analysis of the genotypical information (Fig. 3), we conclude that at both fields the rhizosphere communities correlate well with the bulk soil communities. This observation can be explained by the fact that bacterial rhizosphere colonization is driven by the production of root exudates (Lugtenberg and Dekkers, 1999) to which soil microorganisms are chemo-attracted (Lugtenberg and Kamilova, 2009; Compant *et al*., 2010). Consequently, root exudates control rhizosphere populations like Grayston and colleagues (1998) postulated, but also field-specific soil factors play a significant role since rhizosphere communities at both fields were not identical. Accordingly, Lundberg and colleagues (2012) and Bulgarelli and colleagues (2012) reported that soil type defines the composition of bacterial rhizosphere and root communities of *Arabidopsis thaliana* plants.

A second remarkable observation was that endophytic root communities from both fields were similar (Fig. 3). Lundberg and colleagues (2012) as well as Bulgarelli and colleagues (2012) observed that the host plant determined to a limited extent the bacterial ribotype profiles in roots. We suggest that plants grown from the same seed stock at different fields possess similar obligate endophytes originating from their common seed endophytic community. This statement is based on the exclusive presence of several strains in the roots of plants grown at both fields including *Mycobacterium* (FJ719354), *Pedobacter* (GU385862), *Pseudomonas* (FN377713) and *Variovorax* (GQ861460) (Fig. 1). Other strains exclusively present in the roots at the control field respectively the contaminated field may also be considered as potential seed endophytes since we hypothesize that some of them flourish more than others depending on the local environmental conditions. Root endophytes also isolated from the bulk and/or rhizosphere soil assent with Kobayashi and Palumbo (2000) who mentioned that many endophytic taxa also occur in the rhizosphere. However, in our study, no significant correlation was found between root endophytic and rhizosphere communities within the same field, despite the fact that eight root endophytic strains at the control field and six at the contaminated field were also detected in the rhizosphere soil (Fig. 1). Based on these genotypical data, we presume that plants are capable of favouring the dominance of some specific seed endophytes as obligate endophytes and that the isolated facultative endophytes systemically colonized the inside of the plant via the rhizosphere soil (*cfr.* Compant *et al*., 2010). Probably, most of these facultative endophytes are selected from the soil by plant root exudates that have pronounced selective and promoting effects on specific soil microbial populations (Hartmann *et al*., 2009).

All isolated strains were tested for their Cd and Zn tolerance. The highest numbers of strains tolerant to 1.6 mM Cd and 2.5 mM Zn originated from the contaminated field (Table 2A). Most likely, the significantly higher Cd and Zn concentrations in bulk soil and roots, compared with the background concentrations at the control field (Table 3), caused a selective pressure in favour of Cd and Zn tolerant bacteria at the contaminated field. Moreover, it seems that in this case Zn exerts a higher selective pressure than Cd on bacterial communities since multiple bacterial strains isolated at the contaminated field tolerate the highest Zn concentration, while strains isolated from the control field could hardly survive (Table 2A). Chemical similarity between Cd and Zn and their association in the environment can lead to interactions between these two elements (McKenna *et al*., 1993), resulting in a lowering of Cd toxicity (Wajda *et al*., 1989). Cadmium is, at least partially, taken via the root Zn transporter; therefore high Zn concentrations inhibit Cd uptake and translocation (Polle and Schützendübel, 2003). Further, the uptake and translocation of Zn by plants is higher than Cd (Shrivastava and Singh, 1989) since Cd is a non-essential element and toxic at a lower concentration than Zn (Chakravarty and Shrivastava, 1994).

A high number of bacterial strains isolated from the control rhizosphere and roots could produce siderophores (Table 2B). This might explain why the Fe content in roots of plants grown on the control field was about eight times higher, while the Ca(NO_3_)_2_-extractable Fe concentrations in the soils from both fields were not significantly different (Table 3). Iron deficiency (< 100 mg kg^−1^) was noticed in all parts of plants from the contaminated field; this might inhibit chlorophyll synthesis and chloroplast development and increase ethylene production in plant tissues, eventually leading to decreased remediation efficiency (Glick, 2003). Bacterial siderophore production can promote plant growth, especially in case of iron deficiency by sequestering Fe in siderophore–Fe complexes which plants can use as Fe source (Wang *et al*., 1993). In addition siderophores may also enhance Cd availability since Cd can also be sequestered by siderophores (Rajkumar *et al*., 2010). At both fields the highest amounts of organic acid producing bacteria were found in the rhizosphere (Table 2B). Plant roots exude organic acids into the rhizosphere for the mobilization of poorly soluble nutrients in the soil (Ström *et al*., 2002). An increased acidity in the rhizosphere will also increase TE solubility and eventually phytoextraction potential (Li and Wong, 2010). Most likely, only part of the secreted organic acids will effectively mobilize TEs and nutrients. Further, mobilization is mostly due to both the complexing action of the organic acid anion and the dissolution properties of the released protons. Jones and Darrah (1994) also stated that organic acids can be expected to be of little consequence in high pH soils, while in acid soils (which is the case on the TE-F) they may be involved in a more general uptake mechanism.

At the contaminated field, more bacterial strains showed potential for phosphorus solubilization, nitrogen fixation and production of ACC deaminase and IAA (Table 2B). This might be an indication for bacterial selection by plants during stress conditions. Increased availability of nutrients, bacterial production of plant growth hormones and breakdown of the immediate precursor of the plant stress hormone ethylene can be crucial for plant survival in adverse conditions. In contrast, more acetoin producing bacteria were found at the control field (Table 2B). Higher amounts of organic matter may favour the activity of fermentative bacteria using acetoin as an external energy store (Xiao and Xu, 2007).

This phenotypic information suggests that the TE contamination generates a selective pressure in favour of Cd/Zn tolerant, phosphorus solubilizing, nitrogen fixating and ACC deaminase/IAA producing bacteria since their amounts are consistently higher in all studied compartments at the contaminated field (Table 2).

In conclusion, genotypic and phenotypic characteristics of rapeseed-associated bacterial populations can be affected by environmental conditions (e.g. soil contamination) as well as by their host plant (i.e. selection from the rhizosphere/bulk soil and present seed endophytes). The environmental conditions at the contaminated field seem inductive for the occurrence of rapeseed-associated bacteria with potential to enhance Cd phytoextraction. Enriching the rhizosphere with these PGP, siderophore and/or organic acid producing bacteria might enhance TE uptake while endophytes equipped with a TE sequestration system might reduce Cd phytotoxicity. In future inoculation experiments, the *in planta* potential of promising strains to enhance phytoextraction efficiency will be tested (Sheng and Xia, 2006; Dell'Amico *et al*., 2008; Sheng *et al*., 2008). Once the most appropriate plant-associated bacteria will be identified, they can be exploited to accelerate the TE extraction process, adjusting the high biomass producing *B. napus* into a reasonable Cd phytoextractor that at the same time can be economically valorized.

## Experimental procedures

### Sampling

In order to isolate the cultivable bacteria associated with field grown *B. napus*, soils and plants were sampled after the flowering stage (June 2010). Sampling was performed on a TE (Cd, Zn and Pb)-contaminated former maize field in Lommel (TE-F; see Ruttens *et al*., 2010) and on a non-contaminated field in Alken (Belgium) (CO-F). On both fields the sampling area was subdivided into three subareas. One plant, with its surrounding rhizosphere soil and bulk soil, from each subarea (three in total) made up a mixed bulk soil, rhizosphere soil, root, stem and leaf sample. Sampling was repeated three times using each time three other plants (one per subarea). Bulk soil was sampled at a depth of 30 cm. Roots were stored in sterile Falcon tubes containing 20 ml of sterile 10 mM MgSO_4_.

### Isolation of *B. napus*-associated bacteria

All cultivable bacterial strains were isolated from bulk soil, rhizosphere soil and roots according to Weyens and colleagues (2009c), but using less active chloride solution (1%) and time (1 min) during root surface sterilization. All plated samples were incubated for 7 days at 30°C and colony-forming units (cfu) were counted and calculated per gram soil or fresh plant weight. Morphologically different strains were purified using five replicates and subsequently stored at −70°C in a glycerol solution [15% (w:v) glycerol; 0.85% (w:v) NaCl].

### Genotypic characterization

Total genomic DNA was extracted from all purified morphologically different bacterial strains by the DNeasy® Blood and Tissue kit (Qiagen, Valencia, CA, USA). Polymerase chain reaction (PCR) amplification of the 16S rRNA genes was performed on aliquots of the extracted DNA using the universal primers, 16S-prokaryotic-R (5′-ACGGGCGGTGTGTRC-3′) and 16S-prokaryotic-F (5′-AGAGTTTGATCCTGGCTCAG-3′) as described previously by Weyens and colleagues (2009c).

For amplified 16S rDNA restriction analysis (ARDRA), 20 μl of the PCR products were digested with the HpyCH4IV enzyme and visualized by gel electrophoresis as described by Weyens and colleagues (2009c). Bacterial strains from bulk and rhizosphere soil with the same ARDRA patterns were grouped; strains isolated from plant tissue were grouped separately. The 16S rDNA PCR products of one representative strain per group were purified according to the QIAquick 96 PCR Purification Kit (Qiagen, Valencia, CA, USA). Subsequently, purified 16S rRNA genes were sent for sequencing by Macrogen (Korea) with an Automatic Sequencer 3730XL. Consensus sequences, sequence matches and sequence alignments used for constructing a neighbour-joining tree to verify identification were obtained as in Weyens and colleagues (2009c).

### Phenotypic characterization

All purified bacterial isolates were screened for TE tolerance (Cd and Zn) and potential PGP characteristics (phosphate solubilization, nitrogen fixation and production of siderophores, organic acids, IAA, acetoin and ACC-deaminase). Before screening, strains were grown in 869 medium (Mergeay *et al*., 1985) and subsequently washed twice with sterile 10 mM MgSO_4_. Strains, not able to grow in the test media (pH 7) during incubation [5 (liquid media) to 7 days (solid media) at 30°C], were considered as not detectable (nd). Media without cell suspension were used as controls.

#### TE tolerance

All isolates were plated on selective 284 medium with a carbon mix and 0.0, 0.8 and 1.6 mM Cd (CdSO_4_) or 0.0, 0.6, 1 and 2.5 mM Zn (ZnSO_4_), tolerance was rated visually (Weyens *et al*., 2009c).

#### PGP characteristics

National Botanical Research Institute's phosphate growth solid medium was used for screening **phosphate-solubilizing microorganisms** (Nautiyal, 1999), 50 μl aliquots of washed strains were inoculated in holes (Ø: 0.5 cm). Strains capable of producing a clear zone were considered positive. Bacterial **nitrogenase activity** was tested in a semi-solid malate-sucrose medium modified from Döbereiner (1989) (Xie *et al*., 2006). Three millilitres of bromothymol blue per litre of medium was used as a pH indicator (Nabti *et al*., 2007). Anaerobic nitrogenase activity was visually rated as a colour change from blue to yellow which indicated the acidification of sugars and therefore growth. **Siderophore secretion** was qualitatively evaluated by the ‘universal’ colorimetrical method of Schwyn and Neilands (2010) after inoculating strains in 800 μl of selective 284 medium with a carbon mix and 0, 0.25 and 3 μM Fe (respectively deficient, optimal and oversupply Fe conditions). Bacterial **organic acid production** was detected according to the colorimetric method of Cunningham and Kuiack (1992) after inoculating strains in 800 μl of sucrose tryptone medium. Bacterial **IAA production** capacity was tested in 1 ml of 1/10 869 medium with 0.5 g l^−1^ tryptophan. After incubation, a colorimetric reaction was induced to find positive strains (Gordon and Weber, 1951). To detect strains that utilize the butylene glycol pathway and **produce acetoin**, strains were inoculated in Methyl Red-Voges Proskauer (MRVP) medium containing per litre 17 g of MRVP medium (Sigma-Aldrich). After 48 h of incubation, a colorimetric reaction was induced according to Romick and Fleming (1998), in order to observe positive strains. **ACC deaminase activity** was evaluated by a slight modified protocol according to Belimov and colleagues (2005). Washed bacterial pellets were resuspended in 1 ml salts minimal medium with 10 mM ACC as sole nitrogen source. After 3 days at 30°C, bacterial cells were resuspended in 0.1 ml of Tris-HCl buffer (pH 8.5) and disrupted by 15 μl of toluene. Subsequently, 15 μl of 0.5 M ACC and 100 μl of 0.1 M Tris-HCl buffer (pH 8.5) were added to induce ACC deaminase activity, which was stopped by adding 0.5 ml of 0.56 N HCl. An aliquot of the supernatant was used as described in Belimov and colleagues (2005) to check the presence of ACC deaminase visually.

### Trace element (Na, Mg, K, Fe, Cu, Zn, Cd, Pb) (TE) concentrations in soils and plants

The plant available fractions of TEs present in the bulk soil were estimated using 0.1 M Ca(NO_3_)_2_ extraction (Mench *et al*., 1994). Total soil TE contents were determined by *aqua regia* digestion (Van Ranst *et al*., 1999). To measure total TE concentrations in plant organs (root, stem, leaf and seed), samples collected in the field were treated as described by Weyens and colleagues (2010). TE concentrations were determined using inductively coupled plasma optical emission spectrometry (ICP-OES). All mixed soil and plant samples were tested in triplicate.

### Statistical analysis

Percentages of genotypic and phenotypic different strains per mixed sample and their mean percentages per compartment were calculated but not appropriate for anova analysis. Genotypic information was subjected to correspondence analysis (CA), a principal component analysis related ordination technique based on chi-square distances, illustrating correlations between compartments. A logit transformation was performed on the proportional data gathered during the phenotypic analyses before analysing them using a two-way anova and *post hoc* multiple comparison testing (Tukey Kramer). The same method (without the logit transformation) was used to test the bacterial amounts isolated from the different compartments at both fields. TE concentrations were statistically compared between both fields using one-way anova. Transformations were applied when necessary to approximate normality and/or homoscedasticity. In case normality could not be reached, data were analysed using Kruskal–Wallis multiple comparisons test.

## Supporting Information

Appendix S1 Detailed characterization of all purified bulk soil, rhizosphere soil and *B. napus* root isolates collected at the control field (BS-CO, RS-CO and R-CO respectively) and the contaminated field (BS-TE, RS-TE and R-TE respectively). The presence of each strain is shown as relative abundances, expressed in percentages, of the total number of colony-forming units per gram fresh weight (cfu gFW-1) bulk soil (BS), rhizosphere soil (RS) or roots (R). Strains are identified to the genus level, their accession numbers as well as their presence in the first, second or third replicate (repl) are displayed. Their potential plant growth promoting (PGP) characteristics are indicated by + when positive and by ++(+) in case of a strong positive test. Bacterial strains testing negative for a phenotypic test were labelled by a – symbol and those not applicable for the test by ‘not detected’ (nd). The PGP characteristics tested were Cd (0.8 and 1.6 mM) and Zn (0.6, 1.0 and 2.5 mM) tolerance and the capacity to solubilize phosphorus (P sol), fixate nitrogen (N_2_ fix) and produce siderophores (SID), organic acids (OA), ACC deaminase (ACC), indole-3-acetic acid (IAA) and acetoin.

Appendix S2 The neighbour-joining tree of all strains with a different identification (accession number) clustered strains belonging to the same genus together. Sequences were aligned and used for constructing a neighbour-joining tree with PAUP*4.0b10, using default settings. In order to assess branch supports, bootstrap values were calculated with 2000 pseudoreplicates.

Appendix S3 Total Cd and Zn concentrations {mg [kg dry weight (DW)]_-1_} in roots, stems, leaves and seeds of *B. napus* grown at the control field (CO-F) (A and B respectively) and the contaminated field (TE-F) (C and D respectively). Values are means ± standard error of three biological independent replicates (significance levels: ^*^*P* < 0.05; ^**^*P* < 0.01; ^***^*P* < 0.001; ^****^*P* < 0.0001). Trace element contents measured in different plant parts were compared using two-way ANOVA (plant part and field) and *post hoc* multiple comparison testing (Tukey Kramer).
