# Supplementary material for: Bacterial communities associated with Brassica napus L. grown on trace element-contaminated and non-contaminated fields: a genotypic and phenotypic comparison
Source: Microb Biotechnol. 2013 Apr 18;6(4):371–84. doi: 10.1111/1751-7915.12057 (PMC3917472; doi:10.1111/1751-7915.12057)
Supplement: Appendix S1 — Detailed characterization of all purified bulk soil, rhizosphere soil and B. napus root isolates collected at the control field (BS-CO, RS-CO and R-CO respectively) and the contaminated field (BS-TE, RS-TE and R-TE respectively). The presence of each strain is shown as relative abundances, expressed in percentages, of the total number of colony-forming units per gram fresh weight (cfu gFW-1) bulk soil (BS), rhizosphere soil (RS) or roots (R). Strains are identified to the genus level, their accession numbers as well as their presence in the first, second or third replicate (repl) are displayed. Their potential plant growth promoting (PGP) characteristics are indicated by + when positive and by ++(+) in case of a strong positive test. Bacterial strains testing negative for a phenotypic test were labelled by a – symbol and those not applicable for the test by ‘not detected’ (nd). The PGP characteristics tested were Cd (0.8 and 1.6 mM) and Zn (0.6, 1.0 and 2.5 mM) tolerance and the capacity to solubilize phosphorus (P sol), fixate nitrogen (N2 fix) and produce siderophores (SID), organic acids (OA), ACC deaminase (ACC), indole-3-acetic acid (IAA) and acetoin. [file mbt20006-0371-sd1.doc]

**Appendix 1: Detailed characterisation of all purified bulk soil, rhizosphere soil and *B. napus* root isolates collected at the control field (BS-CO, RS-CO and R-CO respectively) and the contaminated field (BS-TE, RS-TE and R-TE respectively). The presence of each strain is shown as relative abundances, expressed in percentages, of the total number of colony forming units per gram fresh weight (cfu gFW^-1^) bulk soil (BS), rhizosphere soil (RS) or roots (R). Strains are identified to the genus level, their accession numbers as well as their presence in the 1^st^, 2^nd^ or 3^rd^ replicate (repl) are displayed. Their potential plant growth promoting (PGP) characteristics are indicated by + when positive and by ++(+) in case of a strong positive test. Bacterial strains testing negative for a phenotypic test were labeled by a – symbol and those not applicable for the test by ‘not detected’ (nd). The PGP characteristics tested were Cd (0.8 and 1.6 mM) and Zn (0.6, 1.0 and 2.5 mM) tolerance and the capacity to solubilise phosphorus (P sol), fixate nitrogen (N_2_ fix) and produce siderophores (SID), organic acids (OA), ACC deaminase (ACC), indole-3-acetic acid (IAA) and acetoin.**

| BS-CO | | | | | | | | | | | | | | | | | | |
| --- | --- | --- | --- | --- | --- | --- | --- | --- | --- | --- | --- | --- | --- | --- | --- | --- | --- | --- |
| repl | cfu gFW^-1^ | % | identification | | accession | Cd (0.8 mM) | Cd (1.6 mM) | Zn (0.6 mM) | Zn (1 mM) | Zn (2.5 mM) | SID | OA | ACC | IAA | acetoin | P sol | | N_2_ fix |
| 1 | 4783773 | 100.00 | *Micromonospora* | | EU841636 | - | - | - | - | - | - | nd | nd | - | nd | nd | | - |
| 3 | 531237 | 3.51 | *Bacillus* | | AB188212 | - | - | + | + | + | ++ | nd | - | - | +++ | - | | - |
| 3 | 531237 | 3.51 | *Bacillus* | | AB188212 | - | - | - | - | - | - | - | - | - | - | ++ | | - |
| 3 | 53124 | 0.35 | *Bacillus* | | AB188212 | - | - | - | - | - | ++ | - | - | - | +++ | - | | - |
| 3 | 53124 | 0.35 | *Bacillus* | | AB188212 | - | - | - | - | - | + | - | - | - | +++ | - | | - |
| 3 | 212495 | 1.40 | *Bacillus* | | AB188212 | - | - | - | - | - | ++ | - | - | - | +++ | - | | - |
| 3 | 53124 | 0.35 | *Bacillus* | | AB188212 | - | - | - | - | - | ++ | - | - | - | +++ | - | | - |
| 3 | 531237 | 3.51 | *Bacillus* | | CP000813 | - | - | + | + | - | + | + | - | - | +++ | +++ | | - |
| 3 | 531237 | 3.51 | *Bacillus* | | FJ263042 | - | - | + | - | - | + | - | nd | - | ++ | - | | - |
| 3 | 1062473 | 7.01 | *Bacillus* | | FJ263042 | - | - | ++ | - | - | + | - | - | - | +++ | - | | - |
| 3 | 531237 | 3.51 | *Bacillus* | | FJ263042 | - | - | ++ | + | + | + | + | - | - | +++ | ++ | | - |
| 3 | 607128 | 4.01 | *Bacillus* | | FJ263042 | - | - | + | - | - | + | nd | - | - | +++ | ++ | | - |
| 3 | 607128 | 4.01 | *Bacillus* | | FJ263042 | - | - | + | - | - | - | nd | - | - | nd | - | | - |
| 3 | 371866 | 2.45 | *Bacillus* | | FJ263042 | - | - | + | + | + | + | - | - | - | +++ | - | | - |
| 3 | 53124 | 0.35 | *Bacillus* | | FJ263042 | - | - | ++ | + | + | + | + | - | - | +++ | +++ | | - |
| 3 | 53124 | 0.35 | *Bacillus* | | FJ263042 | - | - | + | + | + | + | + | - | - | +++ | +++ | | - |
| 3 | 53124 | 0.35 | *Bacillus* | | FJ263042 | - | - | + | + | + | + | + | - | - | - | - | | - |
| 3 | 5312367 | 35.07 | *Bacillus* | | FJ263042 | - | - | - | - | - | - | - | - | + | - | - | | - |
| 3 | 531237 | 3.51 | *Bacillus* | | GU321095 | - | - | - | - | - | + | ++ | - | - | - | - | | - |
| 3 | 53124 | 0.35 | *Bacillus* | | GU321095 | - | - | - | - | - | + | + | - | - | - | - | | - |
| 3 | 531237 | 3.51 | *Burkholderia* | | FJ786047 | - | - | - | - | - | + | - | - | + | nd | - | | - |
| 3 | 531237 | 3.51 | *Leifsonia* | | AB278552 | - | - | ++ | + | + | - | - | - | + | - | - | | - |
| 3 | 607128 | 4.01 | *Leifsonia* | | AB278552 | - | - | ++ | - | - | - | - | - | + | - | - | | - |
| 3 | 607128 | 4.01 | *Leifsonia* | | AB278552 | - | - | + | - | - | - | - | - | + | - | ++ | | - |
| 3 | 607128 | 4.01 | *Pantoea* | | AF130887 | - | - | + | - | - | + | nd | - | - | +++ | +++ | | ++ |
| 3 | 531237 | 3.51 | *Streptomyces* | | EU119184 | ++ | + | ++ | ++ | + | - | - | - | + | nd | - | | - |
| BS-TE | | | | | | | | | | | | | | | | | | |
| repl | cfu gFW^-1^ | % | identification | | accession | Cd (0.8 mM) | Cd (1.6 mM) | Zn (0.6 mM) | Zn (1 mM) | Zn (2.5 mM) | SID | OA | ACC | IAA | acetoin | P sol | | N_2_ fix |
|  |  |  |  | |  |  |  |  |  |  |  |  |  |  |  |  | |  |
| 1 | 377287 | 1.60 | *Agrobacterium* | | GQ428123 | - | - | + | - | - | + | - | nd | + | nd | nd | | - |
| 1 | 754575 | 3.20 | *Arthrobacter* | | AB288059 | ++ | + | ++ | ++ | ++ | + | + | - | + | - | - | | - |
| 1 | 3772873 | 16.01 | *Arthrobacter* | | AB288059 | - | - | ++ | ++ | ++ | - | - | - | + | - | + | | - |
| 1 | 377287 | 1.60 | *Arthrobacter* | | DQ985470 | - | - | + | - | - | - | - | - | + | - | + | | - |
| 1 | 377287 | 1.60 | *Bacillus* | | AB188212 | - | - | ++ | - | - | + | - | - | - | - | - | | - |
| 1 | 377287 | 1.60 | *Bradyrhizobium* | | FJ390916 | - | - | - | - | - | - | nd | nd | - | nd | nd | | - |
| 1 | 37729 | 0.16 | *Brevundimonas* | | EF088675 | - | - | + | + | + | - | nd | - | - | nd | - | | - |
| 1 | 377287 | 1.60 | *Brevundimonas* | | EF088675 | - | - | ++ | ++ | ++ | + | - | + | - | - | - | | - |
| 1 | 3772873 | 16.01 | *Burkholderia* | | AY949194 | - | - | + | - | - | + | - | - | - | - | - | | - |
| 1 | 377287 | 1.60 | *Burkholderia* | | AY949194 | - | - | + | - | - | - | nd | nd | nd | nd | nd | | - |
| 1 | 377287 | 1.60 | *Burkholderia* | | FJ786047 | - | - | ++ | - | - | - | - | - | - | nd | nd | | nd |
| 1 | 377287 | 1.60 | *Leifsonia* | | AB278552 | - | - | ++ | + | + | - | - | - | + | nd | - | | - |
| 1 | 377287 | 1.60 | *Leifsonia* | | AB278552 | + | - | ++ | - | - | - | - | - | - | - | - | | - |
| 1 | 377287 | 1.60 | *Leifsonia* | | AM889135 | - | - | ++ | ++ | ++ | - | - | - | + | - | + | | + |
| 1 | 754575 | 3.20 | *Leifsonia* | | FJ422386 | - | - | + | + | - | - | - | - | + | - | ++ | | - |
| 1 | 377287 | 1.60 | *Leifsonia* | | FJ422386 | - | - | - | - | - | - | - | + | - | - | ++ | | - |
| 1 | 754575 | 3.20 | *Mesorhizobium* | | AB531422 | - | - | + | + | + | - | - | - | + | nd | + | | - |
| 1 | 471609 | 2.00 | *Methylobacterium* | | Z23158 | - | - | ++ | - | - | + | nd | + | - | - | - | | - |
| 1 | 377287 | 1.60 | *Pedobacter* | | EF660751 | - | - | ++ | + | + | - | - | - | - | - | - | | - |
| 1 | 377287 | 1.60 | *Pseudomonas* | | AB088844 | ++ | + | ++ | - | - | + | - | + | + | - | ++ | | - |
| 1 | 37729 | 0.16 | *Rhodococcus* | | EU496547 | ++ | - | ++ | - | - | + | - | - | - | - | - | | - |
| 1 | 377287 | 1.60 | *Staphylococcus* | | AY167864 | - | - | + | + | + | + | - | + | - | - | ++ | | - |
| 1 | 377287 | 1.60 | unc.bact | | GQ012035 | - | - | ++ | - | - | ++ | - | + | + | - | - | | + |
| 1 | 3772873 | 16.01 | unc.bact | | GQ012035 | - | - | + | + | + | + | nd | + | - | - | - | | - |
| 1 | 3772873 | 16.01 | *Variovorax* | | EF419341 | ++ | + | ++ | - | - | + | - | - | - | - | + | | - |
| 2 | 507924 | 2.33 | *Arthrobacter* | | AB288059 | + | + | ++ | ++ | ++ | - | - | - | + | +++ | + | | - |
| 2 | 50792 | 0.23 | *Arthrobacter* | | AB288059 | + | + | ++ | + | + | - | - | - | + | - | ++ | | + |
| 2 | 507924 | 2.33 | *Arthrobacter* | | AB288059 | - | - | + | - | - | - | - | - | ++ | - | - | | - |
| 2 | 507924 | 2.33 | *Arthrobacter* | | DQ985470 | - | - | + | + | + | + | - | - | ++ | - | - | | - |
| 2 | 50792 | 0.23 | *Bacillus* | | AM910175 | - | - | + | - | - | - | nd | - | + | - | - | | - |
| 2 | 507924 | 2.33 | *Burkholderia* | | FJ786047 | - | - | + | - | - | ++ | - | - | - | - | - | | - |
| 2 | 50792 | 0.23 | *Chryseobacterium* | | DQ337589 | - | - | + | + | + | - | - | - | + | - | - | | - |
| 2 | 672999 | 3.09 | *Leifsonia* | | AB278552 | - | - | ++ | + | + | - | - | - | - | nd | + | | - |
| 2 | 507924 | 2.33 | *Leifsonia* | | AB278552 | - | - | - | - | - | - | - | - | - | - | - | | - |
| 2 | 507924 | 2.33 | *Leifsonia* | | AB278552 | - | - | + | + | + | - | + | - | - | - | - | | - |
| 2 | 507924 | 2.33 | *Leifsonia* | | AM889135 | - | - | ++ | ++ | ++ | - | + | - | + | - | ++ | | - |
| 2 | 50792 | 0.23 | *Leifsonia* | | DQ232613 | - | - | ++ | ++ | + | + | + | - | nd | - | - | | - |
| 2 | 336499 | 1.54 | *Mesorhizobium* | | AY490106 | - | - | + | - | - | + | - | nd | + | - | - | | - |
| 2 | 507924 | 2.33 | *Mesorhizobium* | | AY490106 | - | - | ++ | - | - | - | nd | nd | + | - | ++ | | - |
| 2 | 50792 | 0.23 | *Methylobacterium* | | AB220076 | - | - | + | - | - | nd | - | nd | - | - | - | | - |
| 2 | 50792 | 0.23 | *Methylobacterium* | | Z23158 | - | - | ++ | + | + | + | - | + | + | - | + | | - |
| 2 | 50792 | 0.23 | *Plantibacter* | | AM396918 | - | - | ++ | ++ | ++ | nd | + | - | - | - | - | | - |
| 2 | 50792 | 0.23 | *Pseudomonas* | | FM202488 | + | - | ++ | - | - | + | ++ | + | ++ | - | ++ | | - |
| 2 | 50792 | 0.23 | *Rhodococcus* | | AB425280 | - | - | + | + | + | - | - | - | + | - | - | | - |
| 2 | 507924 | 2.33 | *Rhodococcus* | | EU496547 | + | - | ++ | ++ | ++ | - | - | - | - | - | ++ | | - |
| 2 | 3628026 | 16.65 | *Rhodopseudomonas* | | AB033756 | - | - | + | - | - | nd | - | nd | - | nd | nd | | - |
| 2 | 507924 | 2.33 | *Staphylococcus* | | FJ357589 | - | - | + | + | + | + | - | + | + | - | + | | - |
| 2 | 50792 | 0.23 | *Staphylococcus* | | FJ357589 | - | - | ++ | ++ | ++ | + | - | + | - | - | ++ | | - |
| 2 | 50792 | 0.23 | *Staphylococcus* | | FJ357589 | - | - | ++ | ++ | ++ | + | - | + | - | - | - | | - |
| 2 | 243803 | 1.12 | *Staphylococcus* | | GQ222398 | - | - | ++ | - | - | nd | - | nd | - | nd | nd | | - |
| BS-TE | | | | | | | | | | | | | | | | | | |
| repl | cfu gFW^-1^ | % | identification | | accession | Cd (0.8 mM) | Cd (1.6 mM) | Zn (0.6 mM) | Zn (1 mM) | Zn (2.5 mM) | SID | OA | ACC | IAA | acetoin | P sol | | N_2_ fix |
| 2 | 50792 | 0.23 | *Staphylococcus* | | GQ222398 | - | - | ++ | - | - | + | nd | + | ++ | - | - | | - |
| 2 | 50792 | 0.23 | *Staphylococcus* | | GQ222398 | - | - | ++ | - | - | - | - | - | + | nd | nd | | - |
| 2 | 50792 | 0.23 | *Staphylococcus* | | GQ222398 | - | - | ++ | ++ | ++ | + | + | + | + | - | - | | - |
| 2 | 50792 | 0.23 | *Staphylococcus* | | GQ222398 | - | - | ++ | ++ | ++ | ++ | ++ | + | + | - | - | | - |
| 2 | 5079236 | 23.31 | *Staphylococcus* | | GQ222398 | - | - | ++ | + | + | ++ | - | + | - | - | + | | - |
| 2 | 725605 | 3.33 | *Stenotrophomonas* | | AJ551165 | - | - | ++ | + | + | - | nd | + | - | + | ++ | | - |
| 2 | 507924 | 2.33 | unc.bact | | GQ012035 | - | - | ++ | + | + | + | + | + | + | - | + | | - |
| 2 | 1149177 | 5.27 | unc.bact | | GQ012035 | - | - | ++ | - | - | + | - | + | + | - | - | | + |
| 2 | 121902 | 0.56 | unc.bact | | GQ012035 | - | - | ++ | - | - | + | - | + | - | - | - | | - |
| 2 | 121902 | 0.56 | unc.bact | | GQ012035 | - | - | ++ | - | - | + | - | + | ++ | - | - | | - |
| 2 | 50792 | 0.23 | unc.bact | | GQ012035 | - | - | ++ | + | + | + | - | + | - | - | - | | - |
| 2 | 336499 | 1.54 | unc.bact | | GQ012035 | - | - | ++ | + | + | + | - | + | - | - | - | | - |
| 2 | 336499 | 1.54 | unc.bact | | GQ012035 | - | - | ++ | + | + | + | - | + | + | - | + | | - |
| 2 | 336499 | 1.54 | unc.bact | | GQ012035 | - | - | ++ | - | - | ++ | nd | + | - | - | - | | - |
| 2 | 507924 | 2.33 | unc.bact | | GQ012035 | - | - | ++ | + | + | + | - | + | + | - | - | | - |
| 2 | 507924 | 2.33 | unc.bact | | GQ012035 | - | - | ++ | + | + | + | nd | + | - | - | ++ | | - |
| 2 | 507924 | 2.33 | unc.bact | | GQ012035 | - | - | ++ | + | + | + | + | + | + | - | - | | + |
| 2 | 725605 | 3.33 | unc.bact | | GQ012035 | - | - | ++ | + | + | + | nd | + | ++ | - | - | | - |
| 2 | 50792 | 0.23 | *Variovorax* | | EF419341 | - | - | ++ | - | - | + | - | - | - | - | - | | - |
| 3 | 43463 | 0.27 | *Agrobacterium* | | GQ428123 | ++ | - | ++ | + | - | + | + | + | ++ | - | - | | - |
| 3 | 434631 | 2.70 | *Agrobacterium* | | GQ428123 | - | - | - | - | - | - | - | - | - | - | - | | - |
| 3 | 760605 | 4.72 | *Agrobacterium* | | GQ428123 | - | - | - | - | - | + | - | - | - | - | - | | - |
| 3 | 202828 | 1.26 | *Arthrobacter* | | AB288059 | + | + | + | - | - | + | - | - | - | - | ++ | | nd |
| 3 | 202828 | 1.26 | *Arthrobacter* | | AB288059 | - | - | - | - | - | - | - | - | - | - | - | | - |
| 3 | 202828 | 1.26 | *Arthrobacter* | | AB288059 | - | - | ++ | ++ | ++ | + | - | - | + | - | - | | nd |
| 3 | 43463 | 0.27 | *Arthrobacter* | | AB288059 | + | + | ++ | - | - | - | - | - | - | - | + | | - |
| 3 | 434631 | 2.70 | *Arthrobacter* | | AB288059 | + | + | ++ | + | + | - | - | - | ++ | - | - | | - |
| 3 | 2607789 | 16.17 | *Arthrobacter* | | AB288059 | - | - | ++ | + | + | - | - | - | - | - | - | | - |
| 3 | 434631 | 2.70 | *Arthrobacter* | | AB288059 | - | - | ++ | + | + | - | - | - | - | - | - | | - |
| 3 | 4346314 | 26.95 | *Arthrobacter* | | AB288059 | - | - | ++ | + | + | + | - | + | - | - | ++ | | - |
| 3 | 43463 | 0.27 | *Brevundimonas* | | EF088675 | + | - | ++ | + | + | - | - | + | ++ | - | - | | - |
| 3 | 86926 | 0.54 | *Burkholderia* | | FJ786047 | - | - | ++ | - | - | + | + | - | - | nd | nd | | - |
| 3 | 173853 | 1.08 | *Leifsonia* | | AB278552 | - | - | ++ | - | - | + | nd | nd | - | nd | - | | - |
| 3 | 130389 | 0.81 | *Leifsonia* | | AB278552 | - | - | ++ | ++ | + | - | - | + | + | - | ++ | | - |
| 3 | 2281815 | 14.15 | *Leifsonia* | | AB278552 | - | - | + | - | - | - | - | - | + | - | - | | nd |
| 3 | 434631 | 2.70 | *Leifsonia* | | AB278552 | - | - | + | + | + | - | - | + | + | - | + | | - |
| 3 | 43463 | 0.27 | *Leifsonia* | | AM889135 | - | - | ++ | ++ | ++ | - | + | - | - | - | + | | - |
| 3 | 869263 | 5.39 | *Leifsonia* | | AM889135 | - | - | + | + | - | - | nd | - | - | nd | - | | - |
| 3 | 434631 | 2.70 | *Leifsonia* | | DQ232613 | - | - | ++ | + | + | - | - | + | - | - | ++ | | - |
| 3 | 86926 | 0.54 | *Mycobacterium* | | AY337605 | - | - | + | + | + | + | - | - | - | - | - | | nd |
| 3 | 434631 | 2.70 | *Plantibacter* | | AM396918 | - | - | ++ | ++ | ++ | - | - | - | + | - | ++ | | - |
| 3 | 43463 | 0.27 | *Staphylococcus* | | GQ222398 | - | - | ++ | ++ | ++ | - | - | + | - | - | + | | - |
| 3 | 434631 | 2.70 | unc.bact | | FM872722 | - | - | + | - | - | - | nd | - | - | ++ | - | | - |
| 3 | 434631 | 2.70 | unc.bact | | GQ012035 | - | - | ++ | + | + | - | - | + | + | - | - | | - |
| 3 | 43463 | 0.27 | *Variovorax* | | EF419341 | - | - | ++ | - | - | + | nd | + | ++ | - | - | | - |
| 3 | 434631 | 2.70 | *Variovorax* | | EF419341 | - | - | ++ | + | + | - | - | - | - | - | nd | | - |
| RS-CO | | | | | | | | | | | | | | | | | | |
| repl | cfu gFW^-1^ | % | identification | | accession | Cd (0.8 mM) | Cd (1.6 mM) | Zn (0.6 mM) | Zn (1 mM) | Zn (2.5 mM) | SID | OA | ACC | IAA | acetoin | P sol | | N_2_ fix |
|  |  |  |  | |  |  |  |  |  |  |  |  |  |  |  |  | |  |
|  |  |  |  | |  |  |  |  |  |  |  |  |  |  |  |  | |  |
| 1 | 10599378 | 5.90 | *Aeromicrobium* | | AB245394 | - | - | - | - | - | - | nd | - | - | nd | + | | - |
| 1 | 6838308 | 3.81 | *Agrobacterium* | | GQ428123 | - | - | - | - | - | - | - | - | + | - | ++ | | - |
| 1 | 10257462 | 5.71 | *Bacillus* | | AB188212 | - | - | + | - | - | ++ | - | - | - | +++ | - | | - |
| 1 | 341915 | 0.19 | *Bacillus* | | GU321095 | - | - | - | - | - | + | ++ | - | - | - | - | | ++ |
| 1 | 683831 | 0.38 | *Leifsonia* | | AB278552 | - | - | ++ | - | - | - | - | + | + | - | - | | - |
| 1 | 3419154 | 1.90 | *Leifsonia* | | AB278552 | - | - | - | - | - | - | - | - | + | - | - | | - |
| 1 | 6838308 | 3.81 | *Leifsonia* | | AB278552 | - | - | + | + | - | - | - | - | + | - | ++ | | - |
| 1 | 10599378 | 5.90 | *Lysinibacillus* | | AY907676 | - | - | - | - | - | + | nd | - | - | +++ | - | | - |
| 1 | 13676616 | 7.62 | *Pseudomonas* | | AB369347 | - | - | - | - | - | + | - | + | ++ | - | - | | - |
| 1 | 6838308 | 3.81 | *Pseudomonas* | | AM934699 | - | - | - | - | - | + | - | + | ++ | - | +++ | | - |
| 1 | 3419154 | 1.90 | *Rhodococcus* | | EU496547 | - | - | + | + | + | + | - | - | - | ++ | +++ | | - |
| 1 | 3419154 | 1.90 | *Staphylococcus* | | GQ222398 | - | - | - | - | - | - | nd | - | + | +++ | - | | - |
| 1 | 34191541 | 19.05 | *Staphylococcus* | | GQ222398 | - | - | - | - | - | + | - | nd | - | nd | nd | | - |
| 1 | 34191541 | 19.05 | *Variovorax* | | EF419341 | + | - | - | - | - | + | - | - | - | - | +++ | | - |
| 1 | 34191541 | 19.05 | *Variovorax* | | EF419341 | - | - | - | - | - | - | - | nd | - | ++ | nd | | - |
| 2 | 5439217 | 1.70 | *Agrobacterium* | | GQ428123 | - | - | - | - | - | - | + | - | ++ | - | - | | - |
| 2 | 2472371 | 0.77 | *Agrobacterium* | | GQ428123 | - | - | - | - | - | - | nd | - | + | nd | nd | | - |
| 2 | 2472371 | 0.77 | *Agrobacterium* | | GQ428123 | - | - | - | - | - | - | nd | - | ++ | nd | - | | - |
| 2 | 2472371 | 0.77 | *Arthrobacter* | | AB288059 | - | - | - | - | - | + | - | - | + | - | - | | - |
| 2 | 2472371 | 0.77 | *Arthrobacter* | | AB288059 | - | - | - | - | - | + | - | - | + | - | - | | - |
| 2 | 4944743 | 1.54 | *Arthrobacter* | | AB288059 | - | - | - | - | - | + | - | - | + | - | - | | - |
| 2 | 4944743 | 1.54 | *Arthrobacter* | | AB288059 | - | - | - | - | - | + | - | - | - | - | +++ | | - |
| 2 | 2472371 | 0.77 | *Bacillus* | | AB188212 | - | - | - | - | - | ++ | ++ | - | - | +++ | - | | - |
| 2 | 24723713 | 7.72 | *Bacillus* | | AB188212 | - | - | - | - | - | ++ | - | - | - | ++ | - | | - |
| 2 | 5439217 | 1.70 | *Bacillus* | | AB301017 | - | - | - | - | - | ++ | - | - | - | +++ | - | | - |
| 2 | 2472371 | 0.77 | *Bacillus* | | AJ628743 | - | - | + | - | - | + | - | - | - | - | - | | - |
| 2 | 8900537 | 2.78 | *Bacillus* | | DQ445268 | - | - | + | - | - | + | - | - | + | +++ | + | | - |
| 2 | 7417114 | 2.32 | *Bacillus* | | GQ200827 | - | - | - | - | - | + | + | - | + | - | nd | | - |
| 2 | 2472371 | 0.77 | *Bacillus* | | GQ200827 | - | - | - | - | - | + | - | - | + | nd | +++ | | - |
| 2 | 2472371 | 0.77 | *Bacillus* | | GQ200827 | - | - | - | - | - | + | - | - | + | - | - | | - |
| 2 | 24723713 | 7.72 | *Bacillus* | | GQ200827 | - | - | - | - | - | - | - | - | - | - | - | | - |
| 2 | 4944743 | 1.54 | *Caulobacter* | | DQ337549 | - | - | - | - | - | - | nd | - | - | - | - | | - |
| 2 | 2472371 | 0.77 | *Janthinobacterium* | | D84576 | + | + | ++ | ++ | ++ | + | - | + | + | +++ | ++ | | - |
| 2 | 4944743 | 1.54 | *Kribbella* | | AY253865 | - | - | + | - | - | - | nd | - | - | nd | nd | | - |
| RS-CO | | | | | | | | | | | | | | | | | | |
| repl | cfu gFW^-1^ | % | | identification | accession | Cd (0.8 mM) | Cd (1.6 mM) | Zn (0.6 mM) | Zn (1 mM) | Zn (2.5 mM) | SID | OA | ACC | IAA | acetoin | P sol | N_2_ fix | |
| 2 | 2966846 | 0.93 | | *Labrys* | DQ337554 | - | - | - | - | - | nd | nd | - | ++ | nd | nd | nd | |
| 2 | 4944743 | 1.54 | | *Leifsonia* | AB278552 | - | - | + | - | - | - | - | - | + | - | +++ | - | |
| 2 | 4944743 | 1.54 | | *Leifsonia* | AB278552 | - | - | - | - | - | - | - | - | - | - | - | - | |
| 2 | 2472371 | 0.77 | | *Lysinibacillus* | DQ333300 | - | - | ++ | - | - | - | - | - | + | - | +++ | +++ | |
| 2 | 12361856 | 3.86 | | *Pseudomonas* | AB369347 | + | + | + | - | - | ++ | - | - | + | - | ++ | +++ | |
| 2 | 8653299 | 2.70 | | *Pseudomonas* | AB369347 | + | + | - | - | - | + | - | - | ++ | - | - | - | |
| 2 | 2472371 | 0.77 | | *Pseudomonas* | AB369347 | - | - | ++ | + | + | ++ | ++ | + | + | - | +++ | ++ | |
| 2 | 2472371 | 0.77 | | *Pseudomonas* | AB369347 | - | - | ++ | - | - | + | - | + | + | - | - | - | |
| 2 | 59336910 | 18.53 | | *Pseudomonas* | AM934699 | - | - | - | - | - | + | - | + | ++ | - | - | - | |
| 2 | 5439217 | 1.70 | | *Pseudomonas* | AM934699 | + | - | - | - | - | + | ++ | + | ++ | - | - | - | |
| 2 | 5439217 | 1.70 | | *Pseudomonas* | AM934699 | + | - | - | - | - | + | ++ | + | ++ | - | - | ++ | |
| 2 | 5439217 | 1.70 | | *Pseudomonas* | AM934699 | + | - | - | - | - | + | ++ | + | ++ | - | - | - | |
| 2 | 8653299 | 2.70 | | *Pseudomonas* | AM934699 | + | - | - | - | - | + | - | + | ++ | - | - | ++ | |
| 2 | 8653299 | 2.70 | | *Pseudomonas* | AM934699 | - | - | - | - | - | + | - | + | ++ | - | - | - | |
| 2 | 24723713 | 7.72 | | *Pseudomonas* | AM934699 | + | - | - | - | - | + | ++ | + | ++ | - | - | ++ | |
| 2 | 2966846 | 0.93 | | *Pseudomonas* | FJ772042 | - | - | - | - | - | - | - | + | - | +++ | - | - | |
| 2 | 2472371 | 0.77 | | *Pseudomonas* | FM202488 | ++ | ++ | + | - | - | ++ | - | + | - | - | ++ | +++ | |
| 2 | 4944743 | 1.54 | | *Rhodococcus* | DQ060386 | - | - | + | - | - | + | - | - | - | - | - | - | |
| 2 | 2472371 | 0.77 | | *Sphingobacterium* | AJ438176 | - | - | ++ | - | - | + | - | - | + | - | +++ | - | |
| 2 | 2472371 | 0.77 | | *Staphylococcus* | GQ222398 | - | - | - | - | - | - | - | - | + | - | - | - | |
| 2 | 4944743 | 1.54 | | *Staphylococcus* | GQ222398 | - | - | - | - | - | - | - | + | + | - | +++ | - | |
| 2 | 4944743 | 1.54 | | *Staphylococcus* | GQ222398 | - | - | - | - | - | + | - | - | + | - | +++ | - | |
| 2 | 4944743 | 1.54 | | *Variovorax* | FJ772012 | - | - | - | - | - | + | - | - | + | - | - | - | |
| 2 | 2472371 | 0.77 | | *Variovorax* | FJ772012 | + | + | + | - | - | - | - | + | + | - | nd | - | |
| 2 | 2472371 | 0.77 | | *Variovorax* | FJ772012 | - | - | - | - | - | + | - | - | - | - | - | - | |
| 2 | 7417114 | 2.32 | | *Zoogloea* | X74914 | - | - | - | - | - | - | + | - | ++ | - | + | - | |
| 3 | 50842718 | 0.77 | | *Arthrobacter* | EF028242 | - | - | ++ | - | - | - | - | - | ++ | nd | nd | - | |
| 3 | 508427 | 0.01 | | *Bacillus* | AB188212 | - | - | - | - | - | ++ | - | - | - | +++ | - | - | |
| 3 | 508427 | 0.01 | | *Bacillus* | AB188212 | - | - | - | - | - | ++ | - | - | - | +++ | - | - | |
| 3 | 508427 | 0.01 | | *Bacillus* | AB188212 | - | - | - | - | - | ++ | - | - | - | ++ | - | - | |
| 3 | 508427 | 0.01 | | *Bacillus* | FJ263042 | - | - | ++ | - | - | + | + | - | - | - | - | - | |
| 3 | 50842718 | 0.77 | | *Bacillus* | FJ263042 | - | - | + | - | - | + | + | - | - | + | - | - | |
| 3 | 6406182475 | 96.87 | | *Bacillus* | GQ200827 | - | - | ++ | - | - | + | + | - | - | +++ | - | - | |
| 3 | 50842718 | 0.77 | | *Bacillus* | GQ200827 | - | - | + | - | - | + | + | - | - | ++ | ++ | - | |
| 3 | 508427 | 0.01 | | *Bacillus* | GU321095 | - | - | - | - | - | + | + | - | - | - | - | - | |
| 3 | 508427 | 0.01 | | *Pseudomonas* | FM202488 | + | + | - | - | - | - | - | - | ++ | - | +++ | - | |
| 3 | 508427 | 0.01 | | *Rhodococcus* | EU496547 | - | - | ++ | - | - | + | - | - | - | - | - | - | |
| 3 | 50842718 | 0.77 | | *Streptomyces* | AY314782 | - | - | ++ | - | - | + | - | - | - | nd | - | - | |
| RS-TE | | | | | | | | | | | | | | | | | | |
| repl | cfu gFW^-1^ | % | | identification | accession | Cd (0.8 mM) | Cd (1.6 mM) | Zn (0.6 mM) | Zn (1 mM) | Zn (2.5 mM) | SID | OA | ACC | IAA | acetoin | P sol | N_2_ fix | |
|  |  |  | |  |  |  |  |  |  |  |  |  |  |  |  |  |  | |
| 1 | 3238604 | 0.24 | | *Achromobacter* | GQ927161 | ++ | ++ | - | - | - | - | - | - | - | - | - | ++ | |
| 1 | 1619302 | 0.12 | | *Agrobacterium* | GQ428123 | + | - | ++ | ++ | ++ | + | - | + | + | - | +++ | ++ | |
| 1 | 2267023 | 0.17 | | *Arthrobacter* | AB288059 | - | - | ++ | ++ | + | + | - | - | + | - | + | ++ | |
| 1 | 161930208 | 11.90 | | *Arthrobacter* | AB288059 | + | + | ++ | - | - | + | ++ | - | + | - | - | - | |
| 1 | 3238604 | 0.24 | | *Arthrobacter* | FJ890893 | - | - | - | - | - | + | nd | nd | - | nd | nd | - | |
| 1 | 2267023 | 0.17 | | *Arthrobacter* | FJ890893 | - | - | ++ | - | - | - | nd | nd | - | nd | nd | - | |
| 1 | 1619302 | 0.12 | | *Arthrobacter* | FM213390 | ++ | + | ++ | ++ | + | + | - | - | + | - | - | ++ | |
| 1 | 6801069 | 0.50 | | *Arthrobacter* | FM213390 | - | - | + | + | + | - | - | - | + | - | - | ++ | |
| 1 | 2267023 | 0.17 | | *Arthrobacter* | FM213390 | ++ | + | ++ | ++ | + | + | - | - | + | - | - | - | |
| 1 | 1619302 | 0.12 | | *Arthrobacter* | FM213390 | + | - | ++ | ++ | ++ | + | - | - | + | - | + | - | |
| 1 | 1619302 | 0.12 | | *Arthrobacter* | FM213390 | ++ | + | ++ | ++ | + | + | - | - | + | - | - | - | |
| 1 | 161930208 | 11.90 | | *Bacillus* | AB188212 | - | - | + | - | - | + | - | - | - | - | - | ++ | |
| 1 | 1619302 | 0.12 | | *Brevundimonas* | EF088675 | ++ | + | ++ | ++ | ++ | + | - | - | - | - | - | - | |
| 1 | 14573719 | 1.07 | | *Burkholderia* | FJ939284 | - | - | ++ | + | + | + | - | - | - | - | + | ++ | |
| 1 | 2267023 | 0.17 | | *Burkholderia* | FJ939284 | - | - | ++ | ++ | + | + | - | - | - | - | + | - | |
| 1 | 2428953 | 0.18 | | *Frigoribacterium* | AF157479 | - | - | - | - | - | - | - | - | - | - | - | - | |
| 1 | 4318139 | 0.32 | | *Leifsonia* | AB278552 | - | - | ++ | ++ | + | - | - | - | + | - | ++ | - | |
| 1 | 1619302 | 0.12 | | *Leifsonia* | AB278552 | - | - | ++ | ++ | + | - | - | + | - | - | - | - | |
| 1 | 1619302 | 0.12 | | *Leifsonia* | AB278552 | + | - | ++ | ++ | + | - | - | + | - | - | + | - | |
| 1 | 18621974 | 1.37 | | *Leifsonia* | AB278552 | - | - | - | - | - | - | nd | - | - | nd | - | - | |
| 1 | 18621974 | 1.37 | | *Leifsonia* | GU332619 | - | - | ++ | + | + | - | - | - | + | - | - | - | |
| 1 | 161930208 | 11.90 | | *Leifsonia* | GU332619 | - | - | ++ | - | - | - | - | - | - | - | - | - | |
| 1 | 1619302 | 0.12 | | *Lysinibacillus* | AY907676 | - | - | + | + | + | - | - | - | + | - | ++ | - | |
| 1 | 1619302 | 0.12 | | *Massilia* | AM231588 | - | - | - | - | - | - | - | + | + | - | +++ | - | |
| 1 | 19836450 | 1.46 | | *Microbacterium* | EU821338 | + | + | + | + | + | - | - | + | - | - | - | - | |
| 1 | 19836450 | 1.46 | | *Microbacterium* | EU821338 | + | + | + | + | + | + | - | + | + | - | - | - | |
| 1 | 19836450 | 1.46 | | *Microbacterium* | EU821338 | - | - | + | + | + | - | - | + | + | - | - | - | |
| 1 | 19836450 | 1.46 | | *Microbacterium* | EU821338 | - | - | + | + | + | - | - | + | + | - | - | - | |
| 1 | 3238604 | 0.24 | | *Microbacterium* | EU821338 | + | - | ++ | ++ | ++ | + | - | + | - | - | +++ | - | |
| 1 | 1619302 | 0.12 | | *Pedobacter* | AM279216 | - | - | ++ | ++ | ++ | - | - | - | - | - | - | - | |
| 1 | 3238604 | 0.24 | | *Plantibacter* | AM396918 | + | - | + | + | + | - | - | + | + | - | ++ | - | |
| 1 | 4857906 | 0.36 | | *Plantibacter* | AM396918 | - | - | + | + | + | - | - | + | + | - | + | - | |
| 1 | 1619302 | 0.12 | | *Plantibacter* | AM396918 | - | - | ++ | + | + | - | - | + | + | - | ++ | - | |
| 1 | 4534046 | 0.33 | | *Plantibacter* | AM396918 | ++ | ++ | ++ | ++ | ++ | - | - | + | + | - | ++ | ++ | |
| 1 | 485790624 | 35.71 | | *Plantibacter* | AM396918 | - | - | ++ | + | + | - | + | - | - | - | + | - | |
| 1 | 2428953 | 0.18 | | *Pseudoclavibacter* | X77440 | - | - | ++ | - | - | - | - | - | - | - | ++ | - | |
| 1 | 72868594 | 5.36 | | *Pseudomonas* | AB369347 | - | - | ++ | - | - | ++ | - | + | - | - | ++ | - | |
| 1 | 4318139 | 0.32 | | *Pseudomonas* | AB369347 | + | - | + | + | + | ++ | - | - | - | - | ++ | - | |
| 1 | 2267023 | 0.17 | | *Pseudomonas* | AB369347 | - | - | ++ | - | - | + | - | + | + | - | + | - | |
| 1 | 1619302 | 0.12 | | *Pseudomonas* | AB369347 | - | - | + | + | + | ++ | - | + | - | - | - | - | |
| 1 | 1619302 | 0.12 | | *Pseudomonas* | FJ225200 | + | + | + | - | - | ++ | - | + | - | - | +++ | - | |
| RS-TE | | | | | | | | | | | | | | | | | | |
| repl | cfu gFW^-1^ | % | | identification | accession | Cd (0.8 mM) | Cd (1.6 mM) | Zn (0.6 mM) | Zn (1 mM) | Zn (2.5 mM) | SID | OA | ACC | IAA | acetoin | P sol | | N_2_ fix |
| 1 | 1619302 | 0.12 | | *Rhizobium* | AJ389905 | ++ | - | ++ | ++ | ++ | + | - | + | + | - | +++ | | - |
| 1 | 4318139 | 0.32 | | *Rhizobium* | DQ337581 | - | - | ++ | ++ | ++ | - | - | - | + | - | ++ | | - |
| 1 | 1619302 | 0.12 | | *Sphingobacterium* | AJ438176 | - | - | ++ | - | - | - | - | - | - | - | - | | - |
| 1 | 1619302 | 0.12 | | *Sphingobacterium* | AJ438176 | - | - | ++ | + | + | - | - | - | - | - | +++ | | +++ |
| 1 | 18621974 | 1.37 | | *Sphingobacterium* | AJ438176 | - | - | ++ | ++ | ++ | - | - | - | - | - | - | | - |
| 1 | 18621974 | 1.37 | | *Sphingobacterium* | AJ438176 | - | - | ++ | ++ | ++ | - | - | - | - | - | +++ | | ++ |
| 1 | 1619302 | 0.12 | | *Staphylococcus* | GQ222398 | - | - | + | + | + | - | - | + | + | - | +++ | | - |
| 1 | 18621974 | 1.37 | | *Staphylococcus* | GQ222398 | - | - | + | - | - | - | - | - | + | - | - | | - |
| 1 | 16193021 | 1.19 | | unc.bact | EU536446 | - | - | + | + | + | + | - | + | ++ | - | - | | ++ |
| 1 | 18621974 | 1.37 | | *Variovorax* | EF419341 | - | - | + | - | - | - | - | - | - | - | nd | | - |
| 1 | 1619302 | 0.12 | | *Variovorax* | FJ772012 | - | - | + | - | - | - | - | - | - | - | - | | - |
| 1 | 3238604 | 0.24 | | *Variovorax* | FJ772012 | - | - | + | + | + | + | - | - | - | - | - | | - |
| 1 | 3238604 | 0.24 | | *Variovorax* | FJ772012 | ++ | ++ | - | - | - | - | - | + | - | - | + | | ++ |
| 2 | 7340796 | 2.38 | | *Flavobacterium* | GU078570 | - | - | ++ | ++ | ++ | - | - | - | - | - | ++ | | - |
| 2 | 36703982 | 11.89 | | *Leifsonia* | AB278552 | - | - | ++ | + | + | - | - | - | - | - | ++ | | ++ |
| 2 | 36703982 | 11.89 | | *Leifsonia* | AB278552 | - | - | ++ | + | + | + | + | - | - | - | +++ | | - |
| 2 | 36703982 | 11.89 | | *Leifsonia* | GU332619 | - | - | ++ | + | - | - | - | + | - | - | +++ | | - |
| 2 | 3670398 | 1.19 | | *Methylobacterium* | AB220076 | - | - | ++ | - | - | - | - | - | - | nd | ++ | | - |
| 2 | 36703982 | 11.89 | | *Microbacterium* | EU821338 | - | - | ++ | ++ | ++ | - | - | + | + | - | + | | - |
| 2 | 3670398 | 1.19 | | *Pseudoclavibacter* | X77440 | - | - | + | - | - | - | - | - | - | nd | + | | - |
| 2 | 367040 | 0.12 | | *Staphylococcus* | GQ222398 | - | - | ++ | ++ | ++ | - | - | + | + | - | + | | - |
| 2 | 110111947 | 35.67 | | *Staphylococcus* | GQ222398 | - | - | ++ | ++ | ++ | - | - | + | + | - | - | | ++ |
| 2 | 36703982 | 11.89 | | *Variovorax* | FJ772012 | - | - | ++ | + | + | - | - | - | - | - | +++ | | - |
| 3 | 3480016 | 0.52 | | *Leifsonia* | AB278552 | - | - | ++ | + | - | - | ++ | - | + | - | - | | ++ |
| 3 | 3480016 | 0.52 | | *Leifsonia* | AB278552 | - | - | ++ | ++ | - | - | + | - | + | - | + | | - |
| 3 | 34800160 | 5.15 | | *Leifsonia* | AB278552 | - | - | ++ | ++ | + | - | ++ | - | + | - | ++ | | - |
| 3 | 174000800 | 25.77 | | *Leifsonia* | AB278552 | - | - | ++ | - | - | - | - | - | - | - | + | | - |
| 3 | 34800160 | 5.15 | | *Leifsonia* | AB278552 | - | - | ++ | + | - | - | - | + | - | - | - | | - |
| 3 | 34800160 | 5.15 | | *Pantoea* | EU598802 | - | - | ++ | - | - | + | ++ | - | ++ | +++ | ++ | | - |
| 3 | 3480016 | 0.52 | | *Pseudomonas* | AB369347 | + | + | ++ | + | + | + | - | + | + | - | - | | - |
| 3 | 34800160 | 5.15 | | *Pseudomonas* | AB369347 | - | - | ++ | + | + | + | - | + | - | - | - | | - |
| 3 | 3480016 | 0.52 | | *Rhodococcus* | DQ060386 | + | - | ++ | + | + | - | - | - | - | - | - | | - |
| 3 | 139200640 | 20.62 | | *Serratia* | AJ233434 | + | + | + | + | + | + | - | - | + | +++ | - | | - |
| 3 | 174000800 | 25.77 | | *Staphylococcus* | GQ222398 | - | - | ++ | + | + | - | - | + | - | - | ++ | | + |
| 3 | 34800160 | 5.15 | | *Staphylococcus* | GQ222398 | - | - | ++ | - | - | - | - | - | + | - | - | | - |
| R-CO | | | | | | | | | | | | | | | | | | |
| repl | cfu gFW^-1^ | % | | identification | accession | Cd (0.8 mM) | Cd (1.6 mM) | Zn (0.6 mM) | Zn (1 mM) | Zn (2.5 mM) | SID | OA | ACC | IAA | acetoin | P sol | | N_2_ fix |
| 1 | 3258 | 0.31 | | *Bacillus* | AB188212 | - | - | - | - | - | ++ | - | - | - | +++ | - | | - |
| 1 | 3258 | 0.31 | | *Bacillus* | AJ542508 | + | + | + | - | - | - | - | - | + | - | - | | +++ |
| 1 | 3258 | 0.31 | | *Bacillus* | CP000813 | - | - | - | - | - | + | - | - | - | +++ | - | | - |
| 1 | 14480 | 1.36 | | *Bacillus* | CP000813 | - | - | ++ | + | - | - | nd | - | - | nd | - | | - |
| 1 | 97739 | 9.18 | | *Bacillus* | CP000813 | - | - | ++ | + | + | + | - | - | - | +++ | + | | - |
| 1 | 32580 | 3.06 | | *Bacillus* | CP000813 | - | - | ++ | - | - | + | + | - | - | +++ | - | | - |
| 1 | 3258 | 0.31 | | *Bacillus* | FJ263042 | - | - | + | - | - | + | - | + | - | +++ | +++ | | ++ |
| 1 | 32580 | 3.06 | | *Bacillus* | FJ263042 | - | - | ++ | - | - | + | - | - | - | +++ | ++ | | - |
| 1 | 32580 | 3.06 | | *Bacillus* | FJ263042 | - | - | ++ | - | - | + | - | - | - | +++ | + | | - |
| 1 | 3258 | 0.31 | | *Caulobacter* | DQ337549 | - | - | - | - | - | - | - | - | - | - | +++ | | - |
| 1 | 3258 | 0.31 | | *Caulobacter* | DQ337549 | - | - | - | - | - | - | - | - | + | ++ | - | | - |
| 1 | 3258 | 0.31 | | *Caulobacter* | DQ337549 | - | - | ++ | + | + | - | - | - | - | - | - | | - |
| 1 | 7819 | 0.73 | | *Caulobacter* | DQ337549 | - | - | ++ | - | - | - | - | - | - | +++ | - | | - |
| 1 | 7819 | 0.73 | | *Caulobacter* | DQ337549 | - | - | ++ | - | - | - | - | - | - | - | - | | - |
| 1 | 7819 | 0.73 | | *Caulobacter* | DQ337549 | - | - | - | - | - | - | - | - | - | - | - | | - |
| 1 | 7819 | 0.73 | | *Caulobacter* | DQ337549 | - | - | - | - | - | - | - | - | + | - | ++ | | - |
| 1 | 3258 | 0.31 | | *Caulobacter* | DQ337549 | - | - | - | - | - | - | - | - | - | - | ++ | | - |
| 1 | 6516 | 0.61 | | *Caulobacter* | DQ337549 | - | - | - | - | - | - | nd | - | - | - | ++ | | - |
| 1 | 6516 | 0.61 | | *Caulobacter* | DQ337549 | - | - | + | - | - | - | nd | - | - | - | - | | - |
| 1 | 32580 | 3.06 | | *Caulobacter* | DQ337549 | - | - | - | - | - | - | - | - | - | nd | - | | - |
| 1 | 32580 | 3.06 | | *Caulobacter* | DQ337549 | - | - | - | - | - | - | - | - | - | - | - | | - |
| 1 | 3910 | 0.37 | | *Labrys* | DQ337554 | - | - | ++ | - | - | - | nd | nd | ++ | nd | - | | - |
| 1 | 32580 | 3.06 | | *Labrys* | DQ337554 | - | - | + | - | - | - | nd | nd | - | nd | nd | | - |
| 1 | 32580 | 3.06 | | *Mycobacterium* | FJ719354 | - | - | - | - | - | + | - | - | - | - | - | | - |
| 1 | 26064 | 2.45 | | *Pantoea* | EU598802 | - | - | - | - | - | ++ | - | + | ++ | - | - | | - |
| 1 | 5701 | 0.54 | | *Pseudomonas* | AB330408 | - | - | + | - | - | + | - | - | - | - | + | | - |
| 1 | 19548 | 1.84 | | *Pseudomonas* | AB369347 | + | - | ++ | - | - | + | - | + | + | - | + | | - |
| 1 | 19548 | 1.84 | | *Pseudomonas* | AB369347 | ++ | - | ++ | - | - | + | - | + | + | - | + | | +++ |
| 1 | 3910 | 0.37 | | *Pseudomonas* | DQ778036 | ++ | - | ++ | - | - | + | + | + | + | - | + | | - |
| 1 | 6516 | 0.61 | | *Pseudomonas* | DQ778036 | + | + | - | - | - | + | - | + | ++ | - | - | | - |
| 1 | 3258 | 0.31 | | *Pseudomonas* | DQ778036 | ++ | + | ++ | - | - | + | - | - | - | - | + | | +++ |
| 1 | 6516 | 0.61 | | *Pseudomonas* | DQ778036 | ++ | + | ++ | - | - | + | - | + | + | - | ++ | | - |
| 1 | 3258 | 0.31 | | *Pseudomonas* | FN377713 | ++ | - | ++ | - | - | + | - | + | ++ | - | +++ | | - |
| 1 | 14480 | 1.36 | | *Pseudomonas* | FN377713 | - | - | + | + | + | + | - | + | - | ++ | +++ | | - |
| 1 | 3258 | 0.31 | | *Pseudomonas* | FN377713 | ++ | - | + | + | + | + | - | + | ++ | - | - | | - |
| 1 | 3258 | 0.31 | | *Pseudomonas* | FN377713 | - | - | - | - | - | + | - | + | ++ | - | + | | - |
| 1 | 5701 | 0.54 | | *Pseudomonas* | FN377713 | - | - | + | - | - | + | - | + | ++ | - | +++ | | - |
| 1 | 14480 | 1.36 | | *Rhizobium* | DQ337581 | - | - | + | - | - | - | - | - | - | - | - | | + |
| 1 | 32580 | 3.06 | | *Rhizobium* | DQ337581 | ++ | + | ++ | ++ | + | + | - | - | ++ | - | + | | - |
| 1 | 14480 | 1.36 | | unc.bact | DQ787731 | - | - | - | - | - | - | nd | nd | + | nd | nd | | - |
| 1 | 3910 | 0.37 | | unc.bact | GQ025779 | + | - | ++ | - | - | + | + | - | - | - | ++ | | - |
| 1 | 14480 | 1.36 | | unc.bact | GQ025779 | + | - | + | + | + | - | - | - | - | - | + | | - |
| 1 | 3910 | 0.37 | | *Variovorax* | EF419341 | ++ | - | ++ | ++ | ++ | + | - | - | - | - | - | | - |
| 1 | 14480 | 1.36 | | *Variovorax* | FJ772012 | + | + | ++ | ++ | ++ | + | - | - | - | - | ++ | | ++ |
| R-CO | | | | | | | | | | | | | | | | | | |
| repl | cfu gFW^-1^ | % | | identification | accession | Cd (0.8 mM) | Cd (1.6 mM) | Zn (0.6 mM) | Zn (1 mM) | Zn (2.5 mM) | SID | OA | ACC | IAA | acetoin | P sol | | N_2_ fix |
| 1 | 6516 | 0.61 | | *Variovorax* | GQ861460 | - | - | ++ | ++ | ++ | + | - | - | - | - | - | | - |
| 1 | 3258 | 0.31 | | *Variovorax* | GQ861460 | ++ | - | ++ | ++ | ++ | + | - | - | + | - | - | | - |
| 1 | 3258 | 0.31 | | *Variovorax* | GQ861460 | + | - | ++ | ++ | ++ | + | - | - | - | - | +++ | | - |
| 1 | 26064 | 2.45 | | *Variovorax* | GQ861460 | - | - | - | - | - | + | - | - | - | - | ++ | | - |
| 1 | 32580 | 3.06 | | *Variovorax* | GQ861460 | ++ | - | ++ | + | + | + | - | - | - | - | - | | - |
| 1 | 6516 | 0.61 | | *Variovorax* | GQ861460 | - | - | ++ | - | - | + | - | - | - | - | - | | - |
| 1 | 26064 | 2.45 | | *Variovorax* | GQ861460 | - | - | - | - | - | - | - | - | - | - | - | | - |
| 1 | 6516 | 0.61 | | *Variovorax* | GQ861460 | - | - | + | - | - | + | - | - | - | - | - | | - |
| 1 | 14480 | 1.36 | | *Variovorax* | GQ861460 | - | - | + | - | - | + | - | - | - | - | +++ | | - |
| 1 | 14480 | 1.36 | | *Variovorax* | GQ861460 | - | - | + | - | - | + | - | - | - | - | - | | - |
| 1 | 3258 | 0.31 | | *Variovorax* | GQ861460 | - | - | + | - | - | + | - | - | - | - | - | | - |
| 1 | 15638 | 1.47 | | *Variovorax* | GQ861460 | - | - | + | - | - | + | - | - | - | - | - | | - |
| 1 | 5213 | 0.49 | | *Variovorax* | GQ861460 | - | - | - | - | - | + | - | - | - | - | - | | - |
| 1 | 5213 | 0.49 | | *Variovorax* | GQ861460 | - | - | - | - | - | + | - | - | - | - | - | | - |
| 1 | 3258 | 0.31 | | *Variovorax* | GQ861460 | - | - | - | - | - | + | - | - | - | - | +++ | | - |
| 1 | 3258 | 0.31 | | *Variovorax* | GQ861460 | - | - | ++ | - | - | + | - | - | - | - | +++ | | - |
| 1 | 3258 | 0.31 | | *Variovorax* | GQ861460 | + | - | ++ | + | + | + | - | - | - | - | - | | - |
| 1 | 6516 | 0.61 | | *Variovorax* | GQ861460 | - | - | - | - | - | + | - | - | - | - | ++ | | - |
| 1 | 6516 | 0.61 | | *Variovorax* | GQ861460 | - | - | - | - | - | + | - | - | - | - | - | | - |
| 1 | 5701 | 0.54 | | *Variovorax* | GQ861460 | - | - | + | - | - | + | - | - | - | - | + | | - |
| 1 | 5701 | 0.54 | | *Variovorax* | GQ861460 | - | - | - | - | - | + | - | - | - | - | + | | - |
| 1 | 32580 | 3.06 | | *Variovorax* | GQ861460 | ++ | - | ++ | - | - | + | - | - | - | - | - | | - |
| 1 | 32580 | 3.06 | | *Variovorax* | GQ861460 | - | - | - | - | - | + | - | - | - | - | - | | - |
| 1 | 32580 | 3.06 | | *Variovorax* | GQ861460 | - | - | + | - | - | - | - | - | - | - | - | | - |
| 1 | 32580 | 3.06 | | *Variovorax* | GQ861460 | - | - | + | - | - | + | - | - | - | - | ++ | | - |
| 1 | 32580 | 3.06 | | *Variovorax* | GQ861460 | - | - | + | - | - | + | - | - | - | - | - | | - |
| 1 | 32580 | 3.06 | | *Variovorax* | GQ861460 | - | - | - | - | - | + | - | - | - | - | +++ | | - |
| 1 | 32580 | 3.06 | | *Variovorax* | GQ861460 | - | - | - | - | - | + | - | - | - | - | - | | - |
| 2 | 94153 | 6.55 | | *Bacillus* | CP000813 | - | - | ++ | + | + | + | - | - | - | +++ | + | | - |
| 2 | 47077 | 3.27 | | *Bacillus* | FJ263042 | - | - | + | - | - | ++ | - | - | - | - | - | | - |
| 2 | 4708 | 0.33 | | *Caulobacter* | DQ337549 | - | - | - | - | - | - | - | - | - | - | + | | - |
| 2 | 18831 | 1.31 | | *Labrys* | DQ337554 | - | - | - | - | - | - | nd | - | ++ | nd | - | | - |
| 2 | 4708 | 0.33 | | *Labrys* | DQ337554 | - | - | - | - | - | - | nd | - | ++ | nd | - | | - |
| 2 | 7061 | 0.49 | | *Microbacterium* | DQ328319 | - | - | + | - | - | - | + | - | + | - | +++ | | - |
| 2 | 4708 | 0.33 | | *Paenibacillus* | EU723825 | - | - | + | - | - | - | - | - | + | - | ++ | | - |
| 2 | 7061 | 0.49 | | *Pantoea* | EU598802 | ++ | - | + | - | - | + | - | + | + | - | - | | - |
| 2 | 47077 | 3.27 | | *Pedobacter* | GU385862 | - | - | ++ | - | - | + | - | - | - | - | - | | - |
| 2 | 7061 | 0.49 | | *Plantibacter* | AM396918 | - | - | - | - | - | - | - | + | + | - | - | | - |
| 2 | 7061 | 0.49 | | *Plantibacter* | AM396918 | - | - | + | - | - | + | + | + | + | - | ++ | | - |
| 2 | 211844 | 14.73 | | *Pseudomonas* | AB369347 | + | - | ++ | - | - | + | - | + | + | - | + | | - |
| 2 | 211844 | 14.73 | | *Pseudomonas* | AB369347 | ++ | - | ++ | - | - | + | - | + | + | - | + | | +++ |
| 2 | 6277 | 0.44 | | *Pseudomonas* | AB369347 | ++ | - | ++ | - | - | ++ | - | + | + | - | + | | - |
| 2 | 4708 | 0.33 | | *Pseudomonas* | DQ095904 | - | - | - | - | - | + | - | + | ++ | - | + | | - |
| 2 | 4708 | 0.33 | | *Pseudomonas* | DQ095904 | ++ | - | ++ | - | - | ++ | - | + | + | - | + | | - |
| 2 | 4708 | 0.33 | | *Pseudomonas* | DQ095904 | ++ | - | ++ | + | + | + | - | - | - | - | + | | - |
| 2 | 7061 | 0.49 | | *Pseudomonas* | DQ095904 | - | - | + | + | + | - | - | + | + | - | ++ | | - |
| 2 | 7061 | 0.49 | | *Pseudomonas* | DQ095904 | ++ | - | ++ | ++ | ++ | + | - | - | - | - | ++ | | + |
| 2 | 47077 | 3.27 | | *Pseudomonas* | FJ772042 | - | - | - | - | - | - | - | - | - | - | +++ | | - |
| 2 | 4708 | 0.33 | | *Pseudomonas* | FN377713 | ++ | + | - | - | - | + | + | - | ++ | - | ++ | | - |
| 2 | 263629 | 18.33 | | *Variovorax* | FJ772012 | - | - | ++ | - | - | ++ | - | - | - | - | - | | - |
| 2 | 4708 | 0.33 | | *Variovorax* | GQ861460 | + | - | ++ | + | + | + | - | - | - | - | + | | - |
| 2 | 273044 | 18.99 | | *Variovorax* | GQ861460 | + | - | ++ | + | + | ++ | - | - | - | - | - | | - |
| 2 | 6277 | 0.44 | | *Variovorax* | GQ861460 | - | - | + | - | - | + | - | - | - | - | + | | - |
| 2 | 6277 | 0.44 | | *Variovorax* | GQ861460 | - | - | - | - | - | + | - | - | - | - | + | | - |
| 2 | 7061 | 0.49 | | *Variovorax* | GQ861460 | - | - | - | - | - | + | - | - | - | - | ++ | | - |
| 2 | 4708 | 0.33 | | *Variovorax* | GQ861460 | - | - | - | - | - | + | - | - | - | - | ++ | | - |
| 2 | 47077 | 3.27 | | *Variovorax* | GQ861460 | - | - | ++ | - | - | ++ | - | - | - | - | - | | - |
| 2 | 65907 | 4.58 | | *Variovorax* | GQ861460 | - | - | + | - | - | + | - | - | - | - | + | | - |
| 3 | 3303 | 0.53 | | *Bacillus* | AB188212 | - | - | - | - | - | + | - | - | - | +++ | - | | - |
| 3 | 33033 | 5.26 | | *Bacillus* | AB188212 | - | - | - | - | - | + | - | - | - | +++ | - | | - |
| 3 | 3303 | 0.53 | | *Bacillus* | CP000813 | - | - | ++ | - | - | + | + | - | - | +++ | + | | - |
| 3 | 3303 | 0.53 | | *Brevibacillus* | FJ197026 | - | - | - | - | - | ++ | nd | - | + | - | - | | ++ |
| 3 | 3303 | 0.53 | | *Brevibacillus* | FJ197026 | - | - | ++ | - | - | + | - | - | - | - | - | | - |
| 3 | 6276 | 1.00 | | *Caulobacter* | DQ337549 | - | - | - | - | - | - | - | - | - | - | + | | - |
| 3 | 6276 | 1.00 | | *Caulobacter* | DQ337549 | - | - | ++ | - | - | - | - | - | - | - | + | | - |
| 3 | 6276 | 1.00 | | *Caulobacter* | DQ337549 | - | - | - | - | - | - | - | - | - | - | + | | - |
| 3 | 6276 | 1.00 | | *Caulobacter* | DQ337549 | - | - | - | - | - | - | - | - | - | - | + | | - |
| 3 | 6276 | 1.00 | | *Caulobacter* | DQ337549 | - | - | - | - | - | - | - | - | - | - | - | | - |
| 3 | 6276 | 1.00 | | *Caulobacter* | DQ337549 | - | - | - | - | - | + | - | - | ++ | - | - | | - |
| 3 | 6276 | 1.00 | | *Caulobacter* | DQ337549 | - | - | - | - | - | - | - | - | - | - | + | | - |
| 3 | 3303 | 0.53 | | *Caulobacter* | DQ337549 | - | - | - | - | - | - | - | - | - | - | + | | - |
| 3 | 3303 | 0.53 | | *Caulobacter* | DQ337549 | - | - | + | - | - | - | - | - | - | nd | - | | - |
| 3 | 3303 | 0.53 | | *Caulobacter* | DQ337549 | - | - | - | - | - | + | - | - | - | - | - | | - |
| 3 | 33033 | 5.26 | | *Caulobacter* | DQ337549 | - | - | - | - | - | - | + | - | - | - | + | | - |
| 3 | 13213 | 2.10 | | *Labrys* | DQ337554 | - | - | - | - | - | - | - | + | + | - | - | | - |
| 3 | 101080 | 16.10 | | *Pantoea* | EU598802 | - | - | + | - | - | + | - | + | ++ | + | - | | - |
| 3 | 3303 | 0.53 | | *Pantoea* | EU598802 | + | - | ++ | - | - | + | - | - | + | +++ | - | | - |
| 3 | 33033 | 5.26 | | *Pseudomonas* | AB330408 | - | - | - | - | - | - | - | - | - | - | - | | - |
| 3 | 11231 | 1.79 | | *Pseudomonas* | AB369347 | - | - | - | - | - | + | - | - | ++ | - | + | | - |
| 3 | 31381 | 5.00 | | *Pseudomonas* | AB369347 | + | - | ++ | - | - | + | - | + | + | - | + | | - |
| 3 | 31381 | 5.00 | | *Pseudomonas* | AB369347 | ++ | - | ++ | - | - | + | - | + | + | - | + | | +++ |
| 3 | 9910 | 1.58 | | *Pseudomonas* | AB369347 | - | - | - | - | - | + | - | + | ++ | - | - | | - |
| R-CO | | | | | | | | | | | | | | | | | | |
| repl | cfu gFW^-1^ | % | | identification | accession | Cd (0.8 mM) | Cd (1.6 mM) | Zn (0.6 mM) | Zn (1 mM) | Zn (2.5 mM) | SID | OA | ACC | IAA | acetoin | P sol | | N_2_ fix |
| 3 | 3303 | 0.53 | | *Pseudomonas* | AY047218  AY047218 | + | - | ++ | - | - | + | - | + | - | - | - | | - |
| 3 | 99098 | 15.78 | | *Pseudomonas* | DQ778036 | - | - | - | - | - | + | + | + | ++ | - | + | | - |
| 3 | 33033 | 5.26 | | *Pseudomonas* | DQ778036 | - | - | - | - | - | + | + | + | ++ | - | ++ | | - |
| 3 | 13213 | 2.10 | | *Pseudomonas* | FJ772042 | - | - | + | - | - | - | nd | + | + | - | + | | - |
| 3 | 6276 | 1.00 | | *Pseudomonas* | FN377713 | ++ | - | ++ | - | - | + | - | - | ++ | - | - | | - |
| 3 | 3303 | 0.53 | | *Pseudomonas* | FN377713 | - | - | - | - | - | + | - | + | ++ | - | +++ | | - |
| 3 | 13213 | 2.10 | | *Stenotrophomonas* | FJ772015 | - | - | ++ | - | - | - | - | + | - | - | - | | - |
| 3 | 6276 | 1.00 | | *Variovorax* | GQ861460 | + | - | ++ | + | + | + | - | - | - | - | + | | - |
| 3 | 30390 | 4.84 | | *Variovorax* | GQ861460 | - | - | ++ | - | - | ++ | - | - | - | - | + | | - |
| 3 | 7928 | 1.26 | | *Variovorax* | GQ861460 | - | - | + | + | - | + | - | - | - | - | + | | - |
| 3 | 3964 | 0.63 | | *Variovorax* | GQ861460 | - | - | - | - | - | + | - | - | - | - | - | | - |
| 3 | 3964 | 0.63 | | *Variovorax* | GQ861460 | - | - | - | - | - | + | - | - | - | - | - | | - |
| 3 | 3303 | 0.53 | | *Variovorax* | GQ861460 | - | - | - | - | - | + | - | - | - | - | - | | - |
| 3 | 3303 | 0.53 | | *Variovorax* | GQ861460 | - | - | + | - | - | + | - | - | - | - | - | | - |
| 3 | 13213 | 2.10 | | *Variovorax* | GQ861460 | - | - | - | - | - | + | - | - | - | - | + | | - |
| 3 | 13213 | 2.10 | | *Variovorax* | GQ861460 | - | - | - | - | - | + | - | - | - | - | - | | - |
| 3 | 3303 | 0.53 | | *Variovorax* | GQ861460 | - | - | - | - | - | - | - | - | - | - | - | | - |
| R-TE | | | | | | | | | | | | | | | | | | |
| repl | cfu gFW^-1^ | % | | identification | accession | Cd (0.8 mM) | Cd (1.6 mM) | Zn (0.6 mM) | Zn (1 mM) | Zn (2.5 mM) | SID | OA | ACC | IAA | acetoin | P sol | | N_2_ fix |
|  |  |  | |  |  |  |  |  |  |  |  |  |  |  |  |  | |  |
|  |  |  | |  |  |  |  |  |  |  |  |  |  |  |  |  | |  |
| 1 | 93738 | 1.51 | | *Flavobacterium* | EU057850 | - | - | + | + | + | - | nd | - | ++ | nd | nd | | - |
| 1 | 9374 | 0.15 | | *Pedobacter* | DQ778037 | - | - | ++ | ++ | ++ | - | - | - | - | - | - | | - |
| 1 | 9374 | 0.15 | | *Pedobacter* | DQ778037 | - | - | ++ | ++ | ++ | - | - | - | - | - | - | | - |
| 1 | 93738 | 1.51 | | *Pedobacter* | DQ778037 | - | - | ++ | ++ | ++ | - | - | - | - | - | + | | +++ |
| 1 | 93738 | 1.51 | | *Pedobacter* | DQ778037 | + | + | ++ | ++ | ++ | - | - | - | - | - | - | | - |
| 1 | 2343457 | 37.76 | | *Pseudomonas* | FN377713 | - | - | - | - | - | + | - | + | ++ | - | - | | - |
| 1 | 2343457 | 37.76 | | *Pseudomonas* | FN377713 | - | - | - | - | - | + | - | + | ++ | - | - | | - |
| 1 | 93738 | 1.51 | | *Pseudomonas* | FN377713 | - | - | + | - | - | + | - | + | ++ | - | - | | - |
| 1 | 93738 | 1.51 | | *Rhizobium* | DQ337581 | - | - | - | - | - | - | nd | - | ++ | nd | nd | | - |
| 1 | 524934 | 8.46 | | *Variovorax* | GQ861460 | ++ | - | ++ | + | + | + | - | - | - | - | - | | - |
| 1 | 131234 | 2.11 | | *Variovorax* | GQ861460 | + | + | ++ | + | + | + | - | - | + | - | - | | - |
| 1 | 93738 | 1.51 | | *Variovorax* | GQ861460 | - | - | ++ | - | - | + | - | - | + | - | - | | - |
| 1 | 93738 | 1.51 | | *Variovorax* | GQ861460 | ++ | + | ++ | - | - | + | - | - | - | - | - | | - |
| 1 | 93738 | 1.51 | | *Variovorax* | GQ861460 | + | + | ++ | - | - | + | - | - | ++ | - | - | | - |
| 1 | 93738 | 1.51 | | *Variovorax* | GQ861460 | ++ | + | ++ | - | - | + | - | - | + | - | +++ | | - |
| 2 | 24719 | 18.18 | | *Caulobacter* | DQ337549 | - | - | + | - | - | - | - | - | - | nd | ++ | | - |
| 2 | 6180 | 4.55 | | *Caulobacter* | DQ337549 | + | + | ++ | - | - | - | + | - | - | nd | +++ | | - |
| 2 | 6180 | 4.55 | | *Caulobacter* | DQ337549 | - | - | + | + | + | - | - | - | - | nd | - | | - |
| 2 | 6180 | 4.55 | | *Caulobacter* | DQ337549 | + | + | + | + | + | - | - | - | - | - | +++ | | - |
| 2 | 6180 | 4.55 | | *Flavobacterium* | AM934662 | + | + | ++ | + | + | - | - | - | - | - | +++ | | - |
| 2 | 6180 | 4.55 | | *Pedobacter* | DQ778037 | + | + | ++ | ++ | ++ | - | - | - | - | - | +++ | | +++ |
| 2 | 6180 | 4.55 | | *Pedobacter* | DQ778037 | + | + | ++ | ++ | ++ | - | - | - | - | - | +++ | | - |
| 2 | 6180 | 4.55 | | *Pedobacter* | GU385862 | - | - | ++ | ++ | ++ | - | nd | nd | + | nd | nd | | - |
| 2 | 61797 | 45.45 | | *Rhizobium* | DQ337581 | - | - | - | - | - | - | + | - | ++ | - | ++ | | - |
| 2 | 6180 | 4.55 | | *Rhodococcus* | EU496547 | ++ | ++ | - | - | - | + | - | - | - | - | - | | - |
| 3 | 8673 | 2.26 | | *Caulobacter* | DQ337549 | - | - | + | - | - | - | - | - | - | nd | ++ | | - |
| 3 | 1489 | 0.39 | | *Caulobacter* | DQ337549 | - | - | ++ | - | - | - | - | - | - | - | + | | - |
| 3 | 2365 | 0.62 | | *Caulobacter* | DQ337549 | - | - | + | - | - | - | - | - | - | - | +++ | | - |
| 3 | 2365 | 0.62 | | *Caulobacter* | DQ337549 | - | - | + | - | - | - | - | - | - | - | + | | - |
| 3 | 2365 | 0.62 | | *Caulobacter* | DQ337549 | - | - | ++ | - | - | - | - | - | ++ | - | + | | - |
| 3 | 2190 | 0.57 | | *Caulobacter* | DQ337549 | - | - | ++ | - | - | - | - | - | - | - | +++ | | - |
| 3 | 14630 | 3.82 | | *Caulobacter* | DQ337549 | - | - | + | - | - | - | - | - | - | - | + | | - |
| 3 | 7885 | 2.06 | | *Caulobacter* | DQ337549 | - | - | ++ | ++ | ++ | - | - | - | - | - | - | | - |
| 3 | 7885 | 2.06 | | *Caulobacter* | DQ337549 | - | - | + | + | + | - | - | - | - | - | +++ | | - |
| 3 | 2190 | 0.57 | | *Labrys* | DQ337554 | + | - | ++ | - | - | - | - | - | + | - | - | | - |
| 3 | 788 | 0.21 | | *Labrys* | DQ337554 | ++ | - | ++ | - | - | + | - | + | ++ | - | - | | - |
| 3 | 14630 | 3.82 | | *Labrys* | DQ337554 | - | - | + | - | - | - | - | + | - | nd | - | | - |
| 3 | 2190 | 0.57 | | *Leifsonia* | AB278552 | - | - | ++ | ++ | ++ | + | - | + | + | - | - | | + |
| 3 | 2190 | 0.57 | | *Leifsonia* | AB278552 | - | - | ++ | - | - | + | - | + | - | - | + | | - |
| 3 | 7885 | 2.06 | | *Mucilaginibacter* | EU747841 | - | - | - | - | - | - | - | - | + | nd | - | | - |
| 3 | 7885 | 2.06 | | *Mycobacterium* | FJ719354 | - | - | ++ | ++ | ++ | + | - | - | - | - | - | | - |
| 3 | 2365 | 0.62 | | *Pantoea* | EU598802 | + | + | ++ | ++ | ++ | + | - | + | + | +++ | - | | + |
| 3 | 1656 | 0.43 | | *Pantoea* | EU598802 | + | - | - | - | - | + | ++ | nd | ++ | nd | +++ | | +++ |
| 3 | 7885 | 2.06 | | *Pedobacter* | DQ778037 | - | - | ++ | ++ | ++ | + | - | - | + | - | ++ | | - |
| 3 | 2190 | 0.57 | | *Pedobacter* | DQ778037 | - | - | ++ | ++ | ++ | - | - | + | - | - | +++ | | + |
| 3 | 14630 | 3.82 | | *Pedobacter* | DQ778037 | - | - | ++ | ++ | ++ | - | - | - | - | - | - | | - |
| 3 | 78846 | 20.59 | | *Pedobacter* | DQ778037 | - | - | ++ | ++ | ++ | - | - | + | ++ | - | +++ | | + |
| 3 | 1489 | 0.39 | | *Plantibacter* | AM396918 | - | - | ++ | ++ | + | - | - | + | + | - | +++ | | - |
| 3 | 4731 | 1.24 | | *Plantibacter* | AM396918 | + | - | - | - | - | + | - | - | - | - | - | | - |
| 3 | 2365 | 0.62 | | *Plantibacter* | AM396918 | - | - | ++ | ++ | ++ | - | - | + | + | - | + | | - |
| 3 | 7885 | 2.06 | | *Plantibacter* | AM396918 | - | - | ++ | ++ | ++ | - | + | - | + | - | +++ | | - |
| 3 | 14630 | 3.82 | | *Polaromonas* | AB245355 | - | - | + | + | + | - | - | - | - | - | - | | - |
| 3 | 788 | 0.21 | | *Pseudomonas* | AB369347 | + | + | ++ | - | - | + | - | + | + | +++ | +++ | | - |
| 3 | 2190 | 0.57 | | *Pseudomonas* | AB369347 | + | - | ++ | - | - | ++ | - | + | ++ | - | + | | +++ |
| 3 | 2478 | 0.65 | | *Pseudomonas* | DQ279324 | + | + | ++ | + | + | + | - | + | + | - | ++ | | ++ |
| 3 | 2478 | 0.65 | | *Pseudomonas* | DQ279324 | + | - | ++ | - | - | ++ | - | + | + | - | +++ | | - |
| 3 | 2478 | 0.65 | | *Pseudomonas* | DQ279324 | + | - | ++ | - | - | + | - | + | + | - | +++ | | - |
| 3 | 2478 | 0.65 | | *Pseudomonas* | DQ279324 | + | - | ++ | - | - | ++ | - | + | + | - | ++ | | ++ |
| 3 | 2478 | 0.65 | | *Pseudomonas* | DQ279324 | + | - | ++ | - | - | + | - | + | + | - | + | | ++ |
| 3 | 2478 | 0.65 | | *Pseudomonas* | DQ279324 | + | - | ++ | + | + | ++ | - | + | + | - | - | | ++ |
| 3 | 2478 | 0.65 | | *Pseudomonas* | FJ719351 | - | - | - | - | - | + | - | - | + | - | +++ | | - |
| 3 | 2365 | 0.62 | | *Pseudomonas* | FJ772042 | - | - | ++ | - | - | - | - | - | - | - | - | | - |
| R-TE | | | | | | | | | | | | | | | | | | |
| repl | cfu gFW^-1^ | % | | identification | accession | Cd (0.8 mM) | Cd (1.6 mM) | Zn (0.6 mM) | Zn (1 mM) | Zn (2.5 mM) | SID | OA | ACC | IAA | acetoin | P sol | | N_2_ fix |
| 3 | 788 | 0.21 | | *Pseudomonas* | FJ772042 | - | - | ++ | + | + | - | - | + | + | - | + | | - |
| 3 | 788 | 0.21 | | *Pseudomonas* | FJ772042 | - | - | ++ | - | - | - | - | + | + | - | - | | - |
| 3 | 14630 | 3.82 | | *Pseudomonas* | FJ772042 | - | - | ++ | ++ | ++ | - | - | - | - | - | - | | - |
| 3 | 14630 | 3.82 | | *Pseudomonas* | FJ772042 | - | - | ++ | - | - | - | - | - | - | - | - | | - |
| 3 | 11590 | 3.03 | | *Pseudomonas* | FN377713 | - | - | - | - | - | + | - | - | ++ | +++ | +++ | | - |
| 3 | 1656 | 0.43 | | *Pseudomonas* | FN377713 | + | - | - | + | + | + | - | - | ++ | +++ | +++ | | ++ |
| 3 | 1656 | 0.43 | | *Pseudomonas* | FN377713 | - | - | - | - | - | + | - | - | ++ | +++ | +++ | | - |
| 3 | 7885 | 2.06 | | *Pseudomonas* | FN377713 | + | + | ++ | + | + | + | ++ | - | ++ | - | ++ | | - |
| 3 | 7885 | 2.06 | | *Pseudomonas* | FN377713 | + | + | + | - | - | + | ++ | - | ++ | +++ | +++ | | - |
| 3 | 788 | 0.21 | | *Rhizobium* | DQ337581 | ++ | + | ++ | ++ | - | - | - | + | - | - | - | | - |
| 3 | 1577 | 0.41 | | *Rhizobium* | DQ337581 | - | - | ++ | ++ | - | + | - | - | - | - | +++ | | - |
| 3 | 2190 | 0.57 | | *Rhizobium* | DQ337581 | - | - | - | - | - | + | - | - | ++ | - | ++ | | - |
| 3 | 2365 | 0.62 | | *Rhodococcus* | EU496547 | ++ | - | ++ | + | + | - | - | - | - | - | +++ | | - |
| 3 | 14630 | 3.82 | | *Rhodococcus* | EU496547 | + | - | - | - | - | - | - | - | - | - | ++ | | - |
| 3 | 14630 | 3.82 | | *Rhodococcus* | EU496547 | + | - | - | - | - | - | - | - | - | - | - | | - |
| 3 | 788 | 0.21 | | *Sanguibacter* | X79452 | - | - | ++ | - | - | - | - | - | - | - | - | | - |
| 3 | 788 | 0.21 | | *Sanguibacter* | X79452 | - | - | ++ | + | + | - | - | - | - | - | - | | - |
| 3 | 2365 | 0.62 | | *Staphylococcus* | GQ222398 | - | - | ++ | + | + | - | - | + | - | - | - | | - |
| 3 | 1577 | 0.41 | | *Staphylococcus* | GQ222398 | - | - | + | - | - | - | - | + | + | - | - | | - |
| 3 | 2190 | 0.57 | | unc.bact | GQ012035 | - | - | ++ | ++ | ++ | - | - | + | + | - | + | | - |
| 3 | 1489 | 0.39 | | *Variovorax* | GQ861460 | - | - | ++ | - | - | + | - | - | - | - | - | | - |
| 3 | 1489 | 0.39 | | *Variovorax* | GQ861460 | + | + | ++ | + | + | + | + | - | - | - | - | | - |
| 3 | 1489 | 0.39 | | *Variovorax* | GQ861460 | + | - | ++ | - | - | + | - | - | - | - | - | | - |
| 3 | 1489 | 0.39 | | *Variovorax* | GQ861460 | + | + | ++ | - | - | - | - | - | - | - | ++ | | - |
| 3 | 1489 | 0.39 | | *Variovorax* | GQ861460 | - | - | ++ | - | - | + | - | - | + | - | + | | - |
| 3 | 1489 | 0.39 | | *Variovorax* | GQ861460 | - | - | ++ | - | - | + | - | - | - | - | - | | - |
| 3 | 1489 | 0.39 | | *Variovorax* | GQ861460 | ++ | + | ++ | - | - | + | - | - | - | - | - | | - |
| 3 | 2365 | 0.62 | | *Variovorax* | GQ861460 | - | - | + | + | + | - | ++ | - | + | ++ | - | | - |
| 3 | 2365 | 0.62 | | *Variovorax* | GQ861460 | ++ | + | ++ | ++ | ++ | + | - | - | - | - | - | | - |
| 3 | 1577 | 0.41 | | *Variovorax* | GQ861460 | ++ | ++ | ++ | ++ | ++ | + | - | - | - | - | - | | - |
| 3 | 3154 | 0.82 | | *Variovorax* | GQ861460 | - | - | + | + | + | - | - | - | - | - | - | | - |
| 3 | 788 | 0.21 | | *Variovorax* | GQ861460 | - | - | ++ | + | + | + | - | - | - | - | - | | - |
| 3 | 788 | 0.21 | | *Variovorax* | GQ861460 | - | - | ++ | ++ | ++ | + | - | - | - | - | - | | - |
| 3 | 788 | 0.21 | | *Variovorax* | GQ861460 | - | - | ++ | ++ | ++ | + | - | - | - | - | ++ | | - |
| 3 | 788 | 0.21 | | *Variovorax* | GQ861460 | - | - | ++ | - | - | - | - | - | + | - | - | | - |
| 3 | 788 | 0.21 | | *Variovorax* | GQ861460 | + | + | ++ | + | + | - | - | - | - | - | - | | - |
| 3 | 788 | 0.21 | | *Variovorax* | GQ861460 | + | + | ++ | - | - | - | ++ | - | - | - | - | | - |
| 3 | 788 | 0.21 | | *Variovorax* | GQ861460 | ++ | + | ++ | ++ | ++ | + | - | - | - | - | - | | - |
| 3 | 788 | 0.21 | | *Variovorax* | GQ861460 | + | + | ++ | ++ | ++ | - | - | - | - | - | +++ | | - |
| 3 | 2365 | 0.62 | | *Xanthomonas* | DQ177466 | - | - | ++ | - | - | - | - | - | + | - | - | | - |
