# Supplementary figures and images for: Bacterial communities associated with Brassica napus L. grown on trace element-contaminated and non-contaminated fields: a genotypic and phenotypic comparison
Source: Microb Biotechnol. 2013 Apr 18;6(4):371–84. doi: 10.1111/1751-7915.12057 (PMC3917472; doi:10.1111/1751-7915.12057)

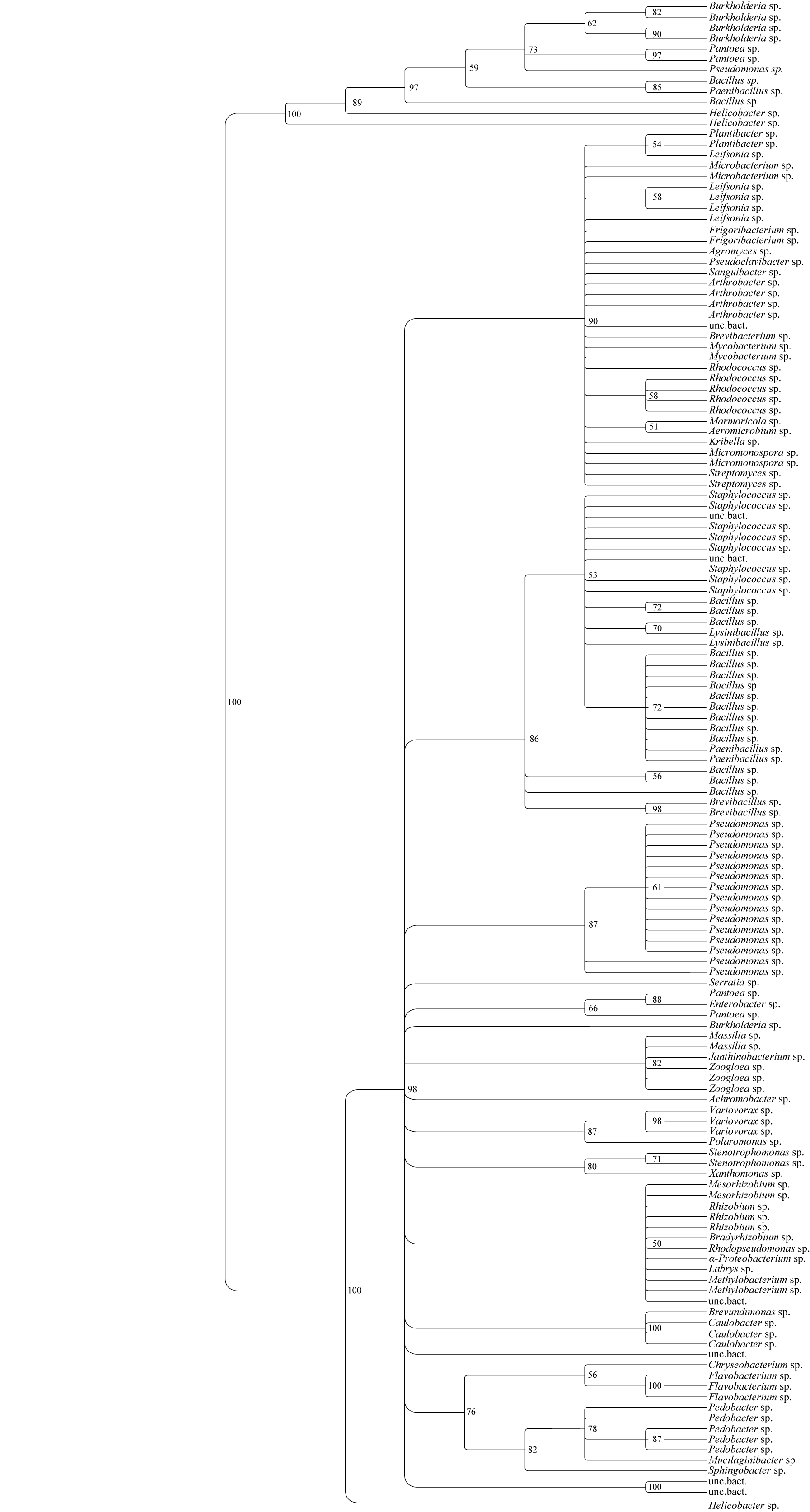

Supplement: Appendix S2 — The neighbour-joining tree of all strains with a different identification (accession number) clustered strains belonging to the same genus together. Sequences were aligned and used for constructing a neighbour-joining tree with PAUP*4.0b10, using default settings. In order to assess branch supports, bootstrap values were calculated with 2000 pseudoreplicates. [file mbt20006-0371-sd2.tif]

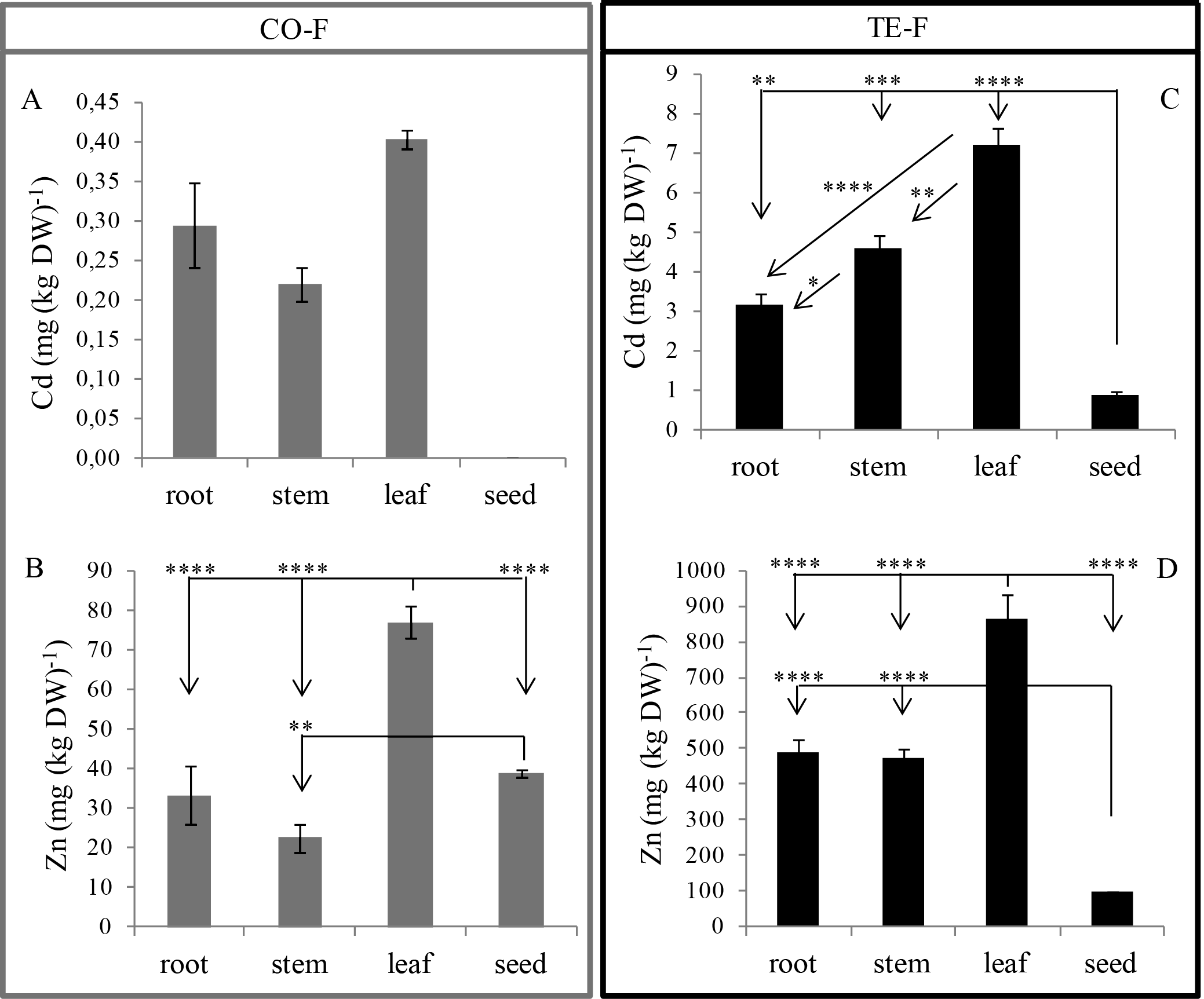

Supplement: Appendix S3 — Total Cd and Zn concentrations {mg [kg dry weight (DW)]-1} in roots, stems, leaves and seeds of B. napus grown at the control field (CO-F) (A and B respectively) and the contaminated field (TE-F) (C and D respectively). Values are means ± standard error of three biological independent replicates (significance levels: *P < 0.05; **P < 0.01; ***P < 0.001; ****P < 0.0001). Trace element contents measured in different plant parts were compared using two-way ANOVA (plant part and field) and post hoc multiple comparison testing (Tukey Kramer). [file mbt20006-0371-sd3.tif]
